# A Ca^2+^-dependent remodelled actin network directs vesicle trafficking to build wall ingrowth papillae in transfer cells

**DOI:** 10.1093/jxb/erx315

**Published:** 2017-09-19

**Authors:** Hui-Ming Zhang, Kim Colyvas, John W Patrick, Christina E Offler

**Affiliations:** 1 School of Environmental and Life Sciences; 2 School of Mathematical and Physical Sciences, The University of Newcastle, Newcastle NSW, Australia

**Keywords:** Actin network, calcium, localized wall deposition, transfer cell, vesicle trafficking, wall ingrowth papillae

## Abstract

The transport function of transfer cells is conferred by an enlarged plasma membrane area, enriched in nutrient transporters, that is supported on a scaffold of wall ingrowth (WI) papillae. Polarized plumes of elevated cytosolic Ca^2+^ define loci at which WI papillae form in developing adaxial epidermal transfer cells of *Vicia faba* cotyledons that are induced to *trans*-differentiate when the cotyledons are placed on culture medium. We evaluated the hypothesis that vesicle trafficking along a Ca^2+^-regulated remodelled actin network is the mechanism that underpins this outcome. Polarized to the outer periclinal cytoplasm, a Ca^2+^-dependent remodelling of long actin bundles into short, thin bundles was found to be essential for assembling WI papillae but not the underlying uniform wall layer. The remodelled actin network directed polarized vesicle trafficking to sites of WI papillae construction, and a pharmacological study indicated that both exo- and endocytosis contributed to assembly of the papillae. Potential candidates responsible for the Ca^2+^-dependent actin remodelling, along with those underpinning polarized exo- and endocyotosis, were identified in a transcriptome RNAseq database generated from the *trans*-differentiating epidermal cells. Of most significance, endocytosis was controlled by up-regulated expression of a dynamin-like isoform. How a cycle of localized exo- and endocytosis, regulated by Ca^2+^-dependent actin remodelling, assembles WI papillae is discussed.

## Teaser

Polarized plumes of elevated cytosolic Ca^2+^ generate a remodelled actin network that directs localized exo- and endocytosis that is responsible for determining the spatially defined assembly of wall ingrowth papillae in transfer cells.

## Introduction

The defining characteristic of transfer cells (TCs) is their wall labyrinth, which forms a superstructure to support an amplified surface area of plasma membrane enriched in membrane transporters that confers an enhanced capacity for nutrient transport (Offler *et al.*, 2003; Andriunas *et al.*, 2013). Reticulate wall labyrinths of TCs are comprised of a uniform wall layer from which wall ingrowth (WI) papillae arise. The inward-directed WI papillae subsequently branch and fuse to form a fenestrated layer of wall material parallel to the uniform wall layer, a process that is repeated to construct the wall labyrinth (Andriunas *et al.*, 2013). To understand the mechanisms and regulatory signals responsible for constructing reticulate wall labyrinths, our investigations have focused on the assembly of the uniform wall layer and the first round of WI papillae formation.

When cotyledons of *Vicia faba* are cultured (Offler *et al.*, 1997), their adaxial epidermal cells rapidly *trans*-differentiate into TCs (Wardini *et al.*, 2007) that are morphologically (Talbot *et al.*, 2001) and functionally (Farley *et al.*, 2000) equivalent to their *in vivo*-formed abaxial counterparts. *Trans*-differentiation to form the wall labyrinth, polarized to the outer periclinal wall, is regulated by a signalling cascade initiated by a culture-induced rise in auxin levels (Dibley *et al.*, 2009). An auxin maximum up-regulates ethylene biosynthesis (Zhou *et al.*, 2010) that in turn promotes a burst in extracellular H_2_O_2_ responsible for switching on deposition of the uniform wall layer (Andriunas *et al.*, 2012; Xia *et al.*, 2012). Co-regulated by ethylene and H_2_O_2_, a Ca^2+^ signal, organized as localized inward-directed plumes of elevated concentrations of cytosolic Ca^2+^ ([Ca^2+^]_cyt_), defines loci at which WI papillae are assembled but exerts no influence on the extracellular reactive oxygen species (ROS)-dependent construction of the polarized uniform wall layer (Andriunas *et al.*, 2012, Xia *et al.*, 2012; Zhang *et al.*, 2015*a*, 2015*c*).

How the Ca^2+^ plumes direct localized deposition of WI papillae is unknown. One key target for [Ca^2+^]_cyt_ regulation of WI papillae formation is through a Ca^2+^ concentration-dependent remodelling of the cytoskeleton to modify the flows of vesicles containing cell wall building materials that are known to play a central role in controlling cell shape (Szymanski and Cosgrove, 2009). However, while the microtubule array of *trans*-differentiating epidermal cells undergoes a [Ca^2+^]_cyt_-dependent remodelling, in common with tip growth of root hairs and pollen tubes (Gu and Nielsen, 2013), WI papillae formation has been found to be microtubule independent (Zhang *et al.*, 2015*b*).

Tip growth of root hairs and pollen tubes is regulated by polarized flow of vesicles along a Ca^2+^-dependent remodelled actin cytoskeleton that is linked with exo- and endocytosis (Gu and Nielsen, 2013). Since construction of WI papillae occurs as a tip-focused phenomenon (Andriunas *et al.*, 2013), this raises the possibility that a similar actin-mediated regulatory mechanism also applies to WI papillae formation. In support of this contention, TC-specific transcripts of a gene encoding actin re-modelling proteins, *Villin-4*, were found to be expressed during WI papillae construction (Zhang *et al.*, 2015*d*). Also detected were transcripts of genes encoding proteins involved in polarized exo- and endocytosis, respectively exocyst complex component *SEC3A-like* and *Dynamin 2B-like*.

Based on the homology of WI papillae construction with tip growth and the TC-specific expression of actin remodelling and vesicle trafficking genes linked with polarized cell development, we tested the hypothesis that Ca^2+^ regulation of WI papillae construction is mediated by polarized vesicle trafficking along a Ca^2+^-dependent remodelled actin network coupled with polarized exocytosis and possibly endocytosis. In the absence of a *V. faba* transformation system, the study relied on pharmacologically perturbing actin remodelling, polarized vesicle trafficking, and exo- and endocytosis of the *trans*-differentiating adaxial epidermal cells and recording the responses of the actin network, vesicle trafficking, and wall labyrinth formation. The cell biology observations were complemented by identifying actin remodelling, vesicle trafficking, and exo- /endocytosis gene transcripts differentially and specifically expressed in *trans*-differentiating epidermal cells. Overall, the findings were consistent with the hypothesis that Ca^2+^-regulated formation of WI papillae was mediated through Ca^2+^ orchestrating the remodelling of the actin network and directing polarized vesicle trafficking.

## Materials and methods

### Plant growth conditions

*Vicia faba* L. cv. Fiord plants were raised under controlled environmental conditions according to Zhou *et al.* (2010).

### Cotyledon culture

Cotyledons (80–120 mg FW per cotyledon) were aseptically cultured on a modified liquid Murashige and Skoog (1962) (MS) medium lacking agar and sucrose (Andriunas *et al.*, 2012), with osmolality adjusted to 315 mOsmol kg^−1^ using betaine (262 mM). Sister cotyledons were divided between MS media either with or without pharmacological agents at specified concentrations (see figure legends and tables for details) and cultured in darkness at 26 °C for 15 h unless stated otherwise. Each pharmacological agent was applied at a concentration that did not negatively impact cell viability, as verified by staining tissue sections of cultured cotyledons with 0.1% (w/v) Tetrazolium Blue.

### Visualization of the actin network and wall ingrowth papillae by confocal laser scanning microscopy

To visualize the actin network, cotyledons were fixed in 2% paraformaldehyde, 5 mM MgCl_2_, 10 mM EGTA, 1% (v/v) glycerol, 0.1% (v/v) TritonX-100 in 0.1 M PIPES (pH 7.0) for 30 min and washed 2 × 5 min in 0.1 M PIPES buffer (pH 7.0) containing 5 mM MgCl_2_ and 10 mM EGTA (protocol modified from Qiao *et al.*, 2010). Paradermal free-hand sections of fixed cotyledon adaxial epidermis were stained with 2 units of Rhodamine-phalloidin (Molecular Probes, Eurgon, USA) for 30 min and excess stain removed by washing sections for 5 min in phosphate-buffered saline (PBS) buffer, pH 7.0. Stained sections were counterstained with 0.1% (w/v) Calcofluor White for 30 s to outline the walls of adaxial epidermal cells before mounting in 200 µl PBS buffer on microscope slides. An Olympus FV 1000 CLSM (confocal laser scanning microscope) with diode-pumped solid-state lasers, combined with an acousto-optic tunable filter (AOTF) laser, was used to visualize the adaxial epidermal cells with a 60× oil immersion objective (NA1.25). A 405-nm UV laser (50 mW, power set to 15%) with a 440–490 nm emission filter set detected the Calcofluor White fluorescence. The focal plane for visualizing the outer periclinal cell wall/cytoplasmic interface was set where the Calcofluor White fluorescence faded. Thereafter, a 559-nm diode laser (15 mW, power set to 25%) with a 550–620 nm emission filter set captured fluorescent images of the Rhodamine-phalloidin-stained actin network at specified *Z* depths from the outer periclinal cell wall/cytoplasmic interface. The images were converted and analysed in FLUOVIEW Viewer 4.0.

**Fig. 1.**
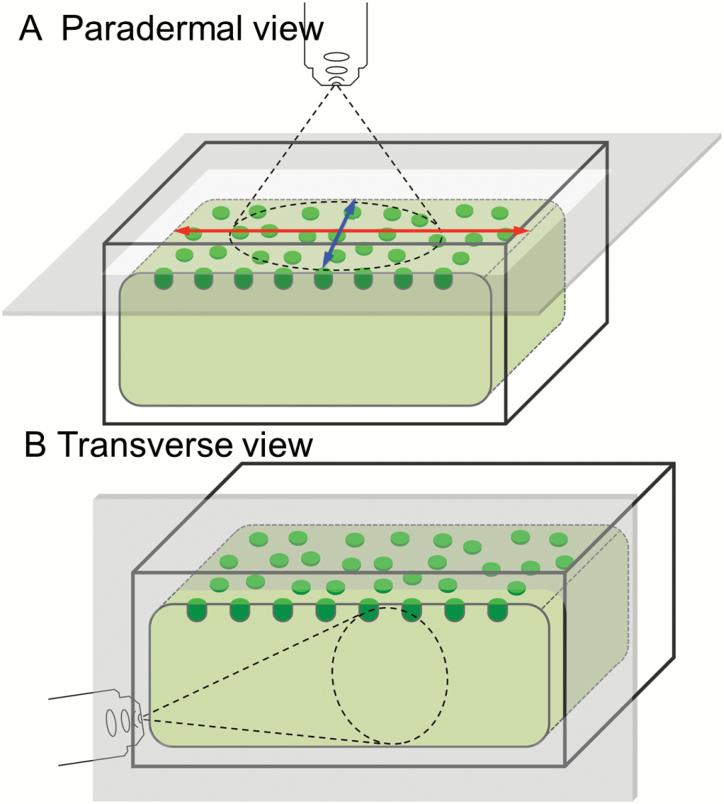
Schematic diagrams of adaxial epidermal cells illustrating the optical planes at which cells were visualized in paradermal (A) and transverse (B) sections. In (A), the long and short axes of the adaxial epidermal cells at their outer periclinal cell wall/cytoplasmic interface are illustrated with red and blue arrows, respectively.

To investigate the spatial relationship between the actin network and WI papillae, paradermal cotyledon sections (Fig. 1A) were stained with 2 units of Alexa-488 phalloidin, as described above, to avoid overlap of emission spectra with that of the cell wall stain, Congo Red. These stained sections were then post-stained with filtered 0.5% (w/v) aqueous Congo Red (Sigma, Australia) for 1 min to visualize WI papillae. A 473-nm diode laser (15 mW, laser power set to 25%) with a 510–550 nm emission filter set captured Alexa-488 phalloidin fluorescence, while a 559-nm diode laser (15 mW, laser power set to 20%) with 610–660 nm emission filter set detected Congo Red. A 60× oil immersion objective (NA1.25) was used to visualize the tissue sections.

### Visualization of the wall labyrinth by transmission and scanning electron microscopy

To assess the impact of the pharmacological agents on formation of the uniform wall layer, ultrathin transverse sections of epidermal cells (Fig. 1B) were visualized with a JEOL 1200 EX II TEM (JOEL, Japan), as previously described (Zhang *et al.*, 2015*b*). Wall thicknesses were estimated using ImageJ software (http://rsbweb.nih.gov/ij/). Given that the thickness of the uniform wall layer is uneven across the outer periclinal wall, the average thickness was estimated from measures of cross-sectional areas divided by their corresponding cell widths (i.e. nm^2^ nm^−1^ = nm). A Phillips XL30 SEM (Phillips, The Netherlands) was used to visualize WI papillae on the cytoplasmic faces of the outer periclinal walls of fractured adaxial epidermal peels, prepared as described in Zhang *et al.* (2015*b*).

### Monitoring vesicle trafficking

To visualize vesicle trafficking in adaxial epidermal cells, transverse free-hand sections (Fig. 1B) were prepared from cotyledons that were either freshly harvested or cultured for 15 h in the absence/presence of specified pharmacological agents. Sections were loaded with 4 μM FM4-64FX (Molecular Probes, Eurgon, USA), an endocytotic marker (Bolte *et al.*, 2004), for 10 min by which time the FM4-64FX fluorescence had reached levels compatible for imaging by CLSM. The loaded sections were mounted in PBS buffer with 100 mM sucrose and placed on microscope slides. A 559-nm diode laser (15 mW, laser power set to 25%) with a 720–770 nm emission filter set (recommended by Molecular Probes) and a 60× oil immersion objective (NA1.25) were used to visualize FM4-64FX fluorescence. The narrow (1 µm) band of cytoplasm in *trans*-differentiating epidermal cells prevented us from distinguishing the fluorescence of FM4-64FX localized to the plasma membrane from that of FM4-64FX-labelled endocytotic vesicles released to the cytoplasm. This problem was mitigated by observing sections of cotyledons incubated on the endo-/exocytotic blocker Brefeldin A (BFA), which restricted FM4-64FX to the plasma membrane. Captured images were converted and analysed in FLUOVIEW Viewer 4.0. Total fluorescence intensities of specified regions of adaxial epidermal cells were measured using ImageJ software corrected for the FM4-64FX fluorescence detected in BFA-treated cells (see Supplementary Table S1 at *JXB* online).

### RNAseq expression analysis of genes related to actin and vesicle trafficking

A previously published *de novo* assembled and validated RNAseq data set, derived from *trans*-differentiating adaxial epidermal and underlying storage parenchyma cells of cultured *V. faba* cotyledons harvested at 0, 3, and 12 h of culture (Zhang *et al.*, 2015*d*), was supplemented with an additional three replicates derived from epidermal peels. The cDNA sequence datasets of raw reads, and the assembled reference transcriptome library supporting the results, are deposited at the European Nucleotide Archive (ENA) with the accession number PRJEB8906 (http://www.ebi.ac.uk/ena/data/view/PRJEB8906). Transcripts were annotated using Blast2GO against the NCBI Genbank (Zhang *et al.*, 2015*d*) and using Mapman Mercator (Lohse *et al.*, 2014) against TAIR, Uniprot, TIGR, KOG and Interpro scan.

Genes encoding proteins known to be involved in actin remodelling and polarized vesicle trafficking, and specifically and differentially expressed in *trans*-differentiating epidermal cells, were identified using the following criteria: mapped reads per kilo base per million reads (RPKM) >1 at 3 or 12 h of cotyledon culture, log_2_-fold change (FC) >1 from 0 to 3 h or 3 to 12 h of cotyledon culture, or sustained up-regulation from 0 to 3 h to 12 h of culture with a false discovery rate (FDR) corrected *P* value <5% determined using LimmaR (see Ritchie *et al.*, 2015). In specified cases, where encoded proteins involved in actin remodelling or vesicle trafficking were known to be Ca^2+^-sensitive, these criteria were relaxed. Functions of encoded proteins were inferred by best-fit percentage amino acid alignment with their closest Arabidopsis homolog. Supplementary Table S2 summarizes bioinformatic details of transcripts identified by the above criteria detected in epidermal and storage parenchyma cells of cultured cotyledons. Supplementary Table S3 lists bioinformatic details of transcripts reported in Zhang *et al.* (2015*d*; see their supplementary table S9) that are absent from Supplementary Table S2 as these transcripts did not satisfy the search criteria.

### Statistical test of spatial correlation between short, thin actin bundles and wall ingrowth papillae

Spatial association between short, thin actin bundles and WI papillae in adaxial epidermal cells was verified statistically. The number and co-ordinates of WI papillae and the number of short, thin actin bundles within the focal plane of the CLSM were recorded in each adaxial epidermal cell that had been co-stained with Congo Red to visualize WI papillae and Alexa-488 phalloidin to visualize actin. The R software package (R Core Team, 2016, V3.3.1) with the library spatstat (Baddeley *et al.*, 2015) was used to run simulations where, using their measured diameter and length, the number of short, thin actin bundles recorded in the cell were laid down in a random pattern over the WI papillae, which were spaced according to their measured co-ordinates. The number of the bundles with their end(s) proximal to WI papillae was recorded, with 1000 simulations performed in each cell. The number of associations from the random simulation data were then compared with those measured from the co-stained cells with significance assessed as single-tail *P*-values determined as the proportion of random simulations yielding an equal or higher percentage of short, thin actin bundles associated with the WI papillae. Note that the criterion of association was determined as the distance between ends of short, thin actin bundles with a distance of <400 nm to the centre of the WI papillae, this being consistent with the association measured from the co-stained cells. A total of 15 cells were tested. Data were expressed as percent of WI papillae associated with end(s) of one or more short, thin actin bundles (see Supplementary Table S4, which includes the R code used for the simulations).

## Results

### Construction of wall ingrowth papillae, but not the uniform wall layer, is dependent on actin

To assess whether the actin network regulated wall labyrinth construction in *trans*-differentiating adaxial epidermal cells, cotyledons were cultured for 15 h in the presence/absence of latrunculin B, which depolymerizes actin (Spector *et al.*, 1983), or jasplakinolide, an actin stabilizer that prevents actin remodelling (Holzinger and Meindl, 1997). To ensure sufficient uptake of the drugs prior to TC *trans*-differentiation commencing, cotyledons were first cultured for 4 h on the ethylene biosynthesis inhibitor, aminoethoxyvinylglycine (AVG) to block TC induction (Zhou *et al.*, 2010) either with or without latrunculin B or jasplakinolide.

**Fig. 2.**
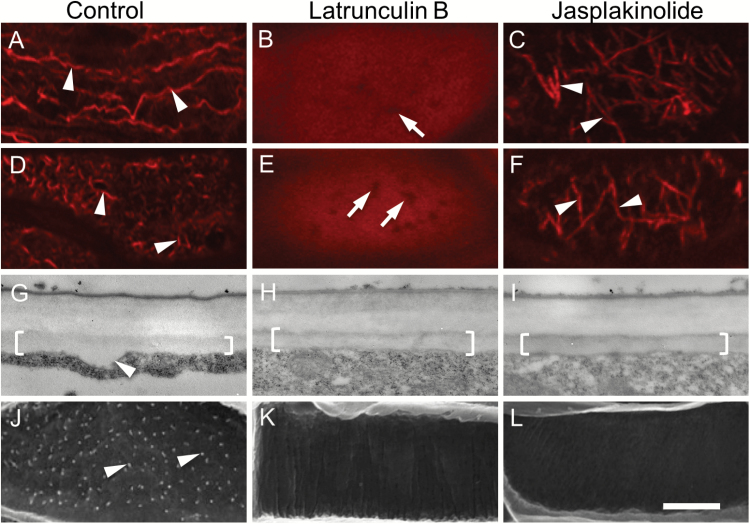
Effect of actin depolymerizing and stabilizing drugs on the actin network, and deposition of the uniform wall layer and WI papillae, in *trans*-differentiating adaxial epidermal cells of cultured *V. faba* cotyledons. Cotyledons were cultured for 4 h in the absence (A, D, G, J) or presence of 100 nM of the actin-depolymerizing drug latrunculin B (B, E, H, K) or 100 nM of the actin-stabilizing drug jasplakinolide (C, F, I, L) together with 100 μM, aminoethoxyvinylglycine (AVG) to inhibit initiation of *trans*-differentiation to a TC morphology (A–C). Thereafter, cotyledons were transferred to AVG-free media and cultured for a further 15 h (D–L). (A–F) Representative CLSM images of the actin network visualized with Rhodamine-phalloidin at the outer periclinal cell wall/cytoplasmic interface (*Z*-depth 0 nm). Comparing (A) with (B) and (C) illustrates the depolymerizing (B) and stabilizing (C) effects of latrunculin B and jasplakinolide on the actin network after the 4-h pre-treatment. By 15 h of culture, following induction of TC *trans*-differentiation, these effects remain unchanged (E, F) except for actin bundles being re-orientated to a more transverse configuration in the jasplakinolide treatment (arrowheads in F). In the absence of the drugs (D), the actin network was remodelled by fragmentation into short actin bundles (arrowheads in D, compare those in A). Actin-free zones (arrows in B, E) are likely to be occupied by mitochondria (1.17 ± 0.03 µm in length; diameter 0.46 ± 0.01 µm; *n*=100). (G–I) Representative TEM images of transverse sections of the outer periclinal wall of adaxial epidermal cells illustrating the uniform wall layer (brackets). The arrowhead in (G) indicates a WI papilla. (J–L) Representative SEM images of the cytoplasmic face of the outer periclinal wall of adaxial epidermal cells. Note WI papillae in (J) (arrowheads) but no papillae in (K) and (L). Scale bar represents 5 μm in (A–F) and 500 nm in (G–I).

At 4 h of culture on AVG, an intact actin network, composed of actin bundles aligned parallel to the long axis of the cell (see Fig. 1A for orientation), was evident in control cells (Fig. 2A). In contrast, in 90% of cells exposed to latruculin B, the actin network was depolymerized, leaving only a smear of actin fluorescence (Fig. 2B, compare with 2A), while in 88% of cells exposed to jasplakinolide, most actin bundles were aligned along the short axis (Fig. 2C, compare with 2A; see Fig. 1A for orientation). This jasplakinolide-induced re-organization of actin bundles has been reported in other plant cells (Cárdenas *et al.*, 2005).

Transferring cotyledons to an AVG-free MS medium for a further 15 h allowed TC *trans*-differentiation to proceed with the construction of the uniform wall layer and WI papillae (Fig. 2G, J). Coincidentally the actin network located at the outer periclinal cell wall/cytoplasmic interface was fragmented into short actin bundles (Fig. 2D, compare with 2A). In contrast, the effects of latrunculin B and jasplakinolide on the actin network, evident after the 4-h treatment, were unchanged (compare Fig. 2B with 2E and Fig. 2C with 2F, respectively). Significantly, WI papillae construction (Table 1; Fig. 2J) was inhibited by latrunculin B and jasplakinolide (Table 1; Fig. 2K, L) while the polarized deposition and thickness of the uniform wall layer was unaffected (Table 1; Fig. 1H, I, compare with 2G). The thicknesses of the anticlinal and inner periclinal walls were also unchanged (Table 1). Collectively, these results were consistent with WI papillae construction being dependent upon a remodelled actin network, while deposition of the uniform wall layer was independent of actin.

**Table 1.** *Effect of actin network remodelling on deposition of the uniform wall layer and WI papillae in* trans*-differentiating adaxial epidermal cells of cultured* V. faba *cotyledons.* Cotyledons were pretreated for 4 h in the presence of the ethylene biosynthesis inhibitor aminoethoxyvinylglycine (AVG) with or without latrunculin B or jasplakinolide. They were then transferred to media without AVG but containing the remaining treatments to induce TC *trans*-differentiation and cultured for a further 15 h. Data are means ±SE. Wall thickness was determined from 15 cells per cotyledon (*n*=90) and percentage of cells with WI papillae was determined from 100 cells per cotyledon using six replicate cotyledons (*n*=6).

Treatment	Thickness (nm)				% cells with WI papillae
	Outer periclinal wall	Anticlinal wall	Inner periclinal wall	Uniform wall layer	
Control	672 ± 18	140 ± 9	192 ± 16	197 ± 4	88 ± 2
Latrunculin B (100 nM)	671 ± 20	132 ± 8	187 ± 15	201 ± 7	22 ± 1
Jasplakinolide (100 nM)	685 ± 16	147 ± 10	200 ± 17	205 ± 6	15 ± 3

### Remodelling of the actin network and construction of wall ingrowth papillae are temporally and spatially correlated

**Fig. 3.**
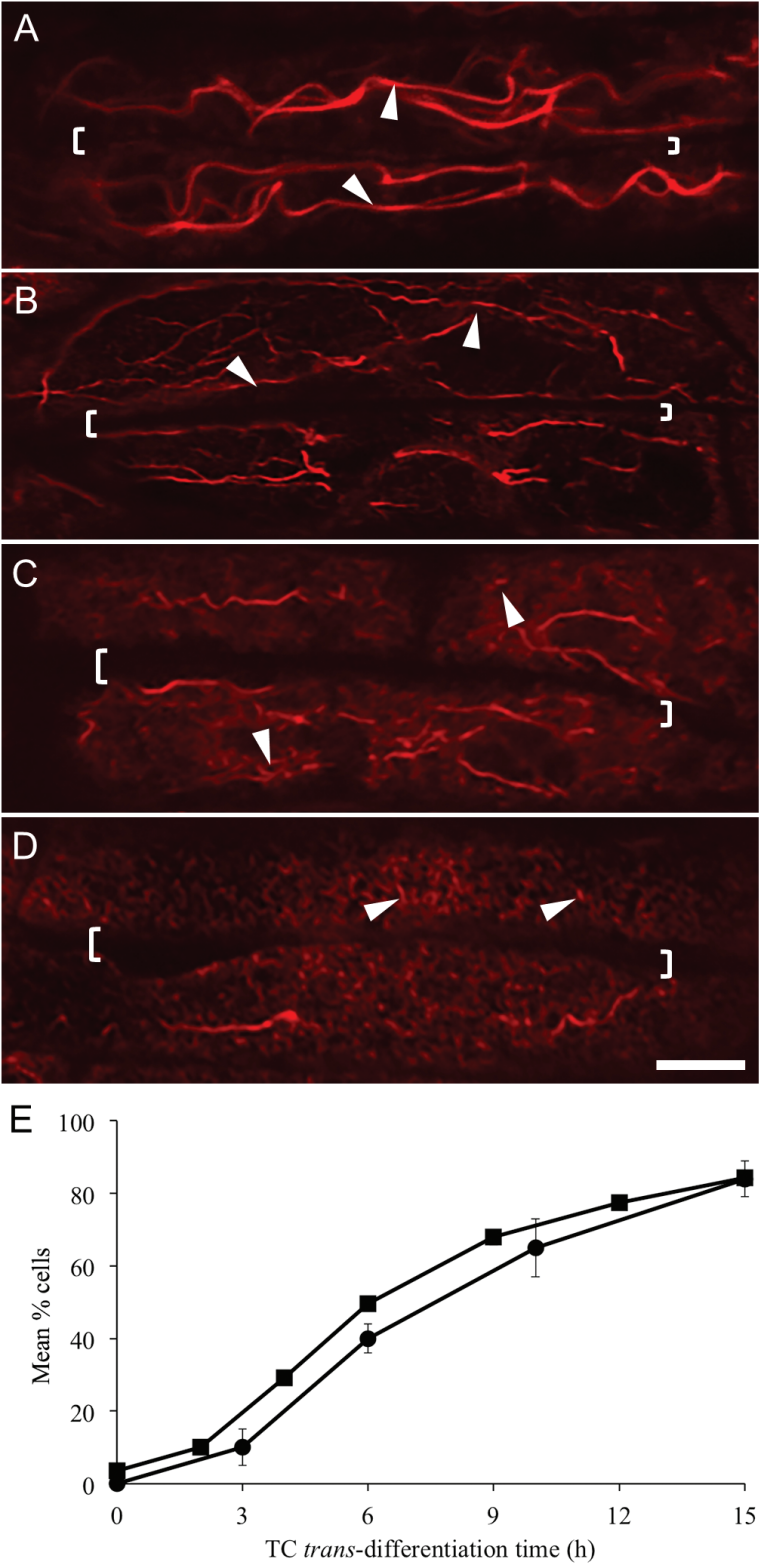
Temporal pattern of actin network remodelling and WI papillae formation in *trans*-differentiating adaxial epidermal cells of cultured *V. faba* cotyledons. (A–D) Representative CLSM images of the actin network visualized with Rhodamine-phalloidin at the outer periclinal cell wall/cytoplasmic interface. The long actin bundles (arrowheads in A and B) aligned parallel to the long axis of the cell (A) become thinner and begin to fragment (B), before progressively fragmenting into short lengths (arrowheads in C, D). Shared walls between two adjoining cells are indicated by square brackets on the images. The scale bar represents 5 μm. (E) Percentage of cells exhibiting a remodelled actin network (squares) or WI papillae (circles; data from Wardini *et al.*, 2007) over a 15-h period of cotyledon culture. An adaxial epidermal cell containing a ‘remodelled network’ was defined as a cell with over 50% of the outer periclinal cell wall/cytoplasmic interface (*Z*-depth 0 nm) being occupied by short actin bundles. Data for actin network remodelling are means ±SE from 100 cells per cotyledon with four replicate cotyledons per time interval (*n*=4).

The progressive temporal change in organization of the actin network was characterized by the long actin bundles becoming thinner (179 ± 3 nm diameter, *n*=120) and beginning to fragment (length 19.6 ± 0.6 µm, n=120; compare Fig. 3A with 3B; Table 2). Subsequent gradual fragmentation (Fig. 3A, compare with 3C) resulted in predominately short, thin actin bundles with an occasional long bundle being detected (compare Fig. 3D with 3A; Table 2). Significantly, percentages of adaxial epidermal cells exhibiting a remodelled actin network across the *trans*-differentiation period were found to follow a temporal profile strongly correlated (*R*^2^>0.98) with that for WI papillae construction (Wardini *et al.*, 2007), the latter being displaced by a lag period of approximately 1 h (Fig. 3E).

**Table 2.** *Actin bundle dimensions* in trans*-differentiating adaxial epidermal cells of cultured* V. faba *cotyledons.* Cotyledons were freshly harvested or cultured for 15 h. Paradermal sections of the cotyledons were stained with Rhodamine-phalloidin and visualized by CLSM at the outer periclinal cell wall/cytoplamic interface (*Z*-depth 0 nm), 500 nm, or 750 nm into the cytoplasm. Note that for 15-h cultured cotyledons differing populations of short actin bundles were detected, namely circular and linear actin bundles at 0 nm *Z*-depth, and linear actin bundles at 500 and 750 nm *Z*-depth. Data are means ±SE of 10 actin bundles per cell, three cells per cotyledon from four replicate cotyledons per treatment (*n*=120). The orientation of actin bundles was expressed as the percentages of total actin bundles scored from 12 cells from four replicate cotyledons (*n*=4).

Culture time (h)	*Z*-depth (nm)	Actin bundle				
		Shape	Length (μm)	Diameter (nm)	Orientation (%)	
					Horizontal	Vertical
0	0	Linear	30.1 ± 0.7	222 ± 7	100 ± 0	0 ± 0
	500	Linear	31.0 ± 0.8	228 ± 10	97 ± 1	3 ± 1
	750	Linear	32.3 ± 0.9	238 ± 11	97 ± 1	3 ± 1
15	0	Linear	1.1 ± 0.1	150 ± 4	89 ± 2	11 ± 2
	0	Circular	1.2 ± 0.1*	152 ± 4	N/A	N/A
	500	Linear	1.0 ± 0.1	157 ± 6	43 ± 3	57 ± 3^**^
	750	Linear	30.6 ± 0.6	225 ± 5	93 ± 2	7 ± 2

* Measured as circumference.

** Diameter of vertical actin bundles: 177 ± 13 nm (*n*=120).

**Fig. 4.**
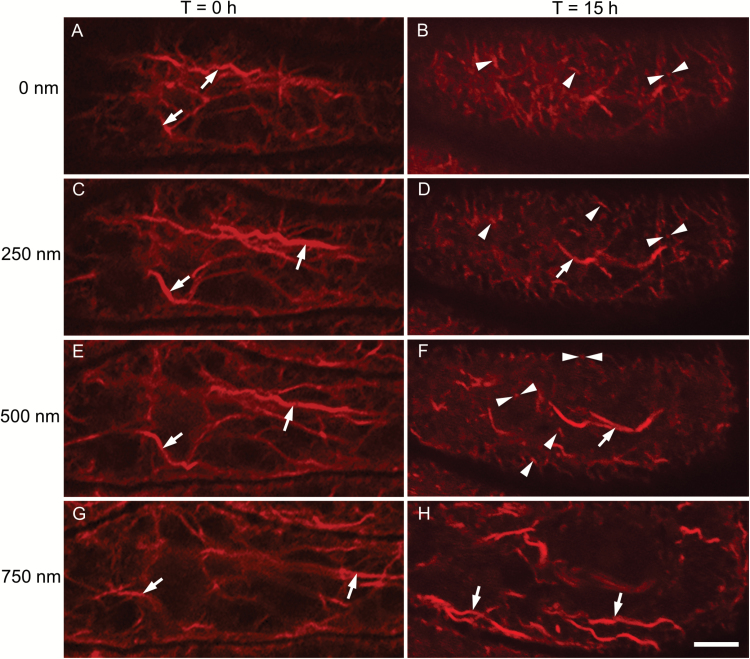
The relationship between the extent of actin bundle severance and distance from the outer periclinal cell wall/cytoplasmic interface in *trans*-differentiating adaxial epidermal cells of cultured *V. faba* cotyledons. The figure shows representative *Z*-stacks of CLSM images of the actin network visualized with Rhodamine-phalloidin in a single adaxial epidermal cell of cotyledons cultured for 0 h (A, C, E, G) or 15 h (B, D, F, H). The focal plane was located at the outer periclinal cell wall/cytoplasmic interface (A, B), i.e. at 0 nm, or inward into the cytoplasm at 250 nm intervals, as indicated. At 0 h of culture, long actin bundles characterized the actin network up to 750 nm into the cytoplasm (arrows in A, C, E, G). After 15 h in culture, short actin bundles were present from the outer periclinal cell wall/cytoplasmic interface (B) to 500 nm (F). These short bundles were linear in shape and oriented either horizontally (single arrowheads in B, D, F) or vertically (paired arrowheads in B, D, F) to the focal plane. A population of longer actin bundles was also evident throughout the cytoplasm to a depth of 500 nm (for example, arrows in D, F). At 750 nm the actin bundles were equivalent in length and diameter to those of 0-h cultured cotyledons (compare H with G). The scale bar represents 5 µm.

To evaluate the spatial relationship between actin remodelling and WI papillae construction, we examined whether actin remodelling was restricted to the depth to which WI papillae protrude into the cytoplasm (i.e. 500 nm; Zhang *et al.*, 2015*b*). To this end, paradermal CLSM images were taken at 250-nm intervals to trace the spatial profile of actin bundle fragmentation deeper into the cytoplasm from the outer periclinal cell wall/cytoplasmic interface (Fig. 4B, D, F, H). Prior to TC induction, long actin bundles, aligned parallel to the longitudinal cell axis, extended throughout the cytoplasm (Fig. 4A, C, E, G). In contrast, after 15 h of culture, actin bundle fragmentation was evident up to, and including, 500 nm deeper into the cytoplasm from the outer periclinal cell wall/cytoplasmic interface (Fig. 4B, D, F). In contrast, at 750 nm there was no evidence of actin bundle fragmentation (Fig. 4H). This analysis indicated that actin network remodelling was confined to the cytoplasmic depth into which WI papillae extend.

**Fig. 5.**
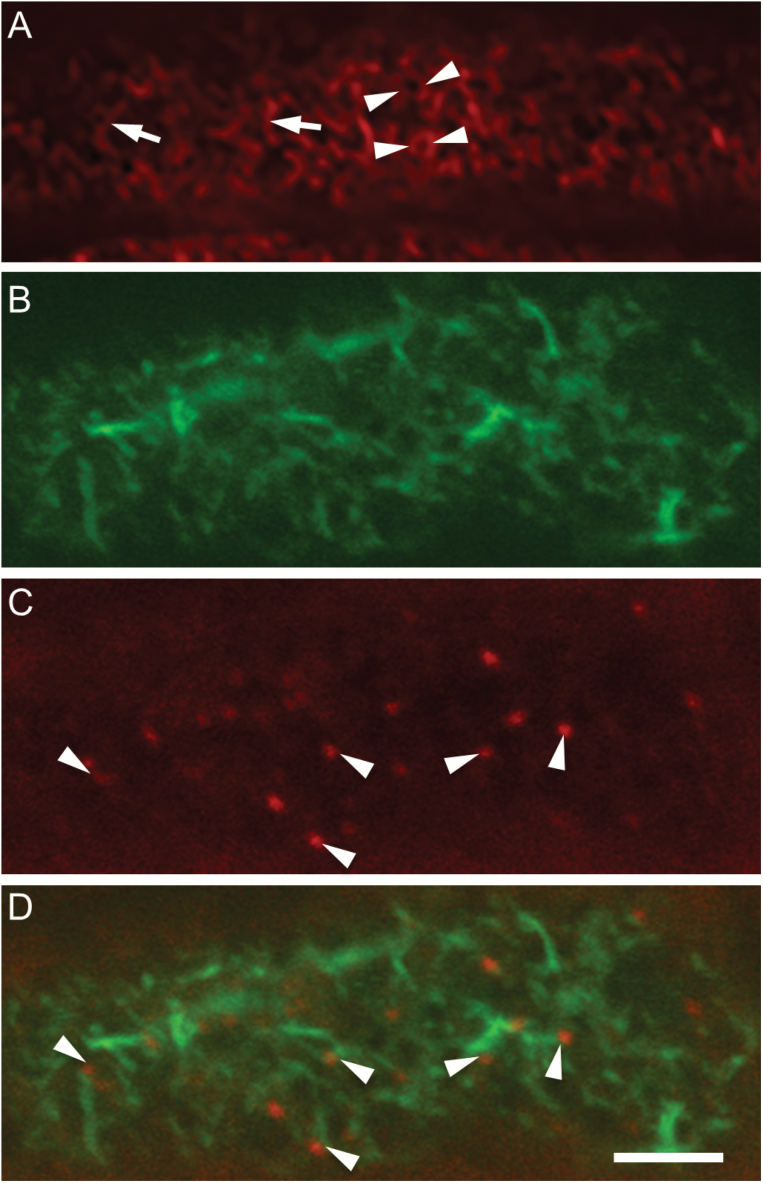
The spatial relationship between the remodelled actin network and developing WI papillae in *trans*-differentiating adaxial epidermal cells of cultured *V. faba* cotyledons. Cotyledons were cultured for 15 h before preparing paradermal sections and staining these with Rhodamine-phalloidin alone or with Alexa-488 phalloidin and Congo Red. Representative CLSM images are shown. (A) The outer periclinal cell wall/cytoplasmic interface of cells stained with Rhodamine-phalloidin alone. Arrowheads indicate actin collars and arrows indicate linear short actin bundles. (B–D) Image at 500 nm inward from the outer periclinal cell wall/cytoplasmic interface, showing (B) the remodelled actin network stained with Alexa-488 phalloidin, (C) WI papillae stained with Congo Red, and (D) a digital overlay of (B) and (C). WI papillae (C, D) are indicated by arrowheads, highlighting their spatial relationship with the linear actin bundles in (D). The scale bar represents 5 µm.

A more detailed examination of the actin network of 15-h cultured cotyledons at their outer periclinal cell wall/cytoplasmic interface identified two populations of short actin bundle shapes, circular and linear (Fig. 5A). Significantly, the inner diameters of circular actin bundles (464 ± 42 nm; *n*=100) corresponded with diameters of WI papillae (Zhang *et al.*, 2015*a*), suggesting that they may form rings around initiating papillae (Fig. 5A). Both types of actin bundles were similar in circumference/length and diameter (Table 2). However, the short linear bundles were possibly located deeper into the cytoplasm than the circular ones (Fig. 5A) and were present up to 500 nm into the cytoplasm (Fig. 4F). At 500 nm, the short linear bundles were of comparable dimensions to those located at the outer periclinal cell wall/cytoplasmic interface (Table 2) and accounted for 83 ± 3% (*n*=50) of the total actin bundle population. At increasing depths into the cytoplasm, the proportions of horizontally to vertically orientated linear bundles (appearing as dots of comparable diameter, Table 2) changed from 89% at the outer periclinal wall/cytoplasmic interface to 43% at 500 nm (Table 2; Fig. 4B, D, F). Another population of longer (7.5 ± 1.5 nm; *n*=120) linear bundles, with diameters equivalent to those of longitudinal bundles prior to culture (195 ± 12; *n*=120, compare with Table 2), accounted for 14–20% of actin bundles up to 500 nm (Fig. 4B, D, F). Deeper into the cytoplasm at 750 nm, the short actin bundles were replaced by long and thick bundles of similar dimensions to those found in epidermal cells prior to culture (Fig. 4H; Table 2).

The spatial relationship between short actin bundles and tips of WI papillae was explored using higher magnification images at the 500-nm focal plane, which was selected to include a high proportion of horizontally oriented actin bundles. Paradermal sections of adaxial epidermal cells were co-stained with Alexa-488 phalloidin (to label actin; Fig. 4B) and Congo Red (to label WI papillae; Fig. 5C). When the two images were overlaid (Fig. 5D), WI papillae appeared to be proximal to ends of one or more of the short, thin actin bundles in the focal plane (distance between centres of WI papillae and ends of actin bundles <400 nm). Indeed, a survey of overlay images indicated that 59.1 ± 1.9% (*n*=50) of WI papillae conformed to this spatial interrelationship. The statistical significance of this was tested by simulation for 15 cells (*n*=1000 simulations per cell; see Methods and Supplementary Table S4 for details). The results showed that such high levels of association were very unlikely to have come about by chance, with *P*-values of 0.004 and 0.001 for two cells and *P*<0.001 for the other 13. Given the significance observed in these 15 randomly selected cells from the 50 sampled, this clearly demonstrates that the spatial association between short, thin actin bundles and WI papillae is beyond random coincidence. The association between WI papillae and actin bundles was reproduced for the outer periclinal cell wall/cytoplasmic interface (71.1 ± 4%; *n*=50).

These findings collectively indicate that, during TC *trans*-differentiation, actin bundles in the outermost periclinal region of adaxial epidermal cells become fragmented and the resulting short actin bundles form a close spatial relationship with WI papillae.

### Actin network remodelling is mediated by plumes of elevated cytosolic Ca^2+^

Since plumes of elevated [Ca^2+^]_cyt_ define loci at which WI papillae form (Zhang *et al.*, 2015*a*), and WI papillae and short actin bundles were spatiotemporally correlated (Figs 2–4), we hypothesized that Ca^2+^ plumes could play a regulatory role in remodelling the actin network. To examine this hypothesis, cotyledons were cultured in the presence of nifedipine, a DHP-receptor Ca^2+^-permeable channel blocker, which attenuates formation of Ca^2+^ plumes by blocking cytosolic polarized entry of extracellular Ca^2+^ into the epidermal cells (Zhang *et al.*, 2015*a*).

**Fig. 6.**
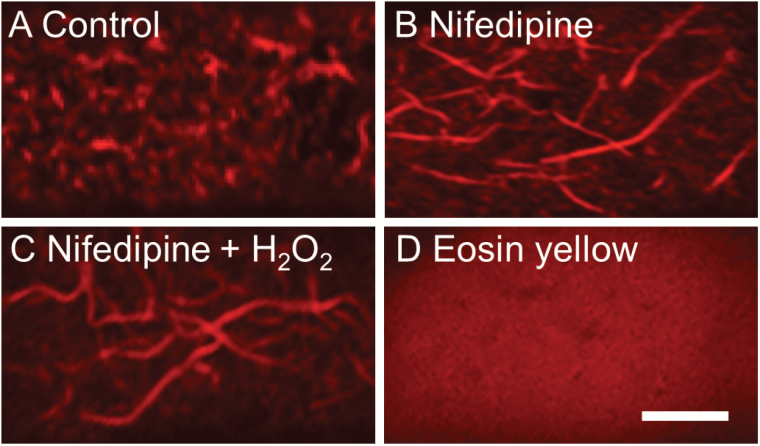
Effect of cytosolic Ca^2+^ plumes on remodelling of the actin network in *trans*-differentiating adaxial epidermal cells of cultured *V. faba* cotyledons. Cotyledons were cultured for 15 h in the absence (A) or the presence of (B) 100 µM of the DHP receptor Ca^2+^-permeable channel blocker nifedipine, (C) 100 µM nifedipine plus 10 µM H_2_O_2_, or (D) 500 nM of the plasma membrane Ca^2+^-ATPase inhibitor eosin yellow. The figure shows representative CLSM images of the actin network stained with Rhodamine-phalloidin, located at the outer periclinal cell wall/cytoplasmic interface. The scale bar represents 5 µm.

Nifedipine halted remodelling of the actin network in 80% of adaxial epidermal cells (compare Fig. 6B with 6A; Table 3). However, this outcome could have resulted from nifedipine attenuating [Ca^2+^]_cyt_-regulated activities of respiratory burst oxidases and producing extracellular reactive oxygen species (ROS; Andriunas *et al.*, 2012), as seen in ROS-induced actin remodelling in pollen tubes and stomata (Wilkins *et al.*, 2011; Li *et al.*, 2014). To address this issue, nifedipine-treated cotyledons were supplemented with an exogenous supply of H_2_O_2_ to ensure that [Ca^2+^]_cyt_ was attenuated whilst WI papillae formation driven by ROS signalling was not affected (Andriunas *et al.*, 2012). Under these conditions, only 15% of cells displayed a remodelled actin network (Fig. 6C; Table 3); a result comparable to that for nifedipine alone (compare Fig. 6C with 6B). These findings demonstrated that remodelling of the actin network was mediated by [Ca^2+^]_cyt_.

**Table 3.** *Effect of Ca* ^*2+*^ *and ROS on actin network remodelling* in trans*-differentiating adaxial epidermal cells of cultured* V. faba *cotyledons* Cotyledons were cultured for 15 h in the absence/presence of the DHP receptor-type Ca^2+^-permeable channel blocker nifedipine, or nifedipine plus exogenous H_2_O_2_. Data are means ±SE determined from 100 cells per cotyledon and four replicate cotyledons (*n*=4).

Treatment	% cells with remodelled actin network
Control	80.0 ± 0.5
Nifedipine (100 μM)	15.1 ± 1.6
Nifedipine (100 μM) + H_2_O_2_ (10 μM)	14.6 ± 0.8

To determine whether actin remodelling depends upon the [Ca^2+^]_cyt_ signal being spatially organized into plumes, these were dissipated by an eosin block of plasma membrane Ca^2+^-ATPase activity that caused an elevated [Ca^2+^]_cyt_ to be spread uniformly throughout the entire cytosol (Zhang *et al.*, 2015*a*). This treatment elicited actin depolymerization in over 95% of adaxial epidermal cells (Fig. 6D), similar to that imposed by latrunculin B (Fig. 2B). This finding supports the conclusion that Ca^2+^ plumes cause localized actin bundle depolymerization and hence their fragmentation.

### A remodelled actin network is required to maintain polarized vesicle trafficking

The temporal and spatial remodelling of the actin network correlated with, and was required for, WI papillae construction (Figs 2–5; Tables 1, 3), suggesting that the actin network may function in providing tracks for polarized and localized vesicle delivery to sites of WI papillae construction. To investigate this possibility, transverse sections of cotyledons, either freshly harvested (0 h, control) or cultured for 15 h in the absence/presence of a vesicle-trafficking inhibitor or actin-manipulating chemicals, were stained with FM4-64FX, a membrane-selective fluorescent dye introduced into cells by labelling endocytotic vesicles (Bolte *et al.*, 2004). To obtain estimates for vesicle fluorescence alone, measured intensity values were corrected for the contribution of fluorescence arising from the plasma membrane using sections of cotyledons cultured on the exo-/endocytotic blocker, BFA (see Supplementary Table S1).

After 15 h of cotyledon culture, overall vesicle trafficking activity, as measured by FM4-64FX fluorescent intensity, was enhanced 4.6-fold relative to that of 0-h control cotyledons (Table 4). This increase was entirely accounted for by the high fluorescence intensity located in the outer periclinal cytoplasm. When actin network remodelling was disrupted by latrunculin B or jasplakinolide, overall fluorescence of FM4-64FX endocytosed into the cytoplasm was unaffected, indicating the endocytosis was independent of actin. However, fluorescence was redistributed around the adaxial epidermal cells, suggesting that the polarized vesicle trafficking observed in control cells was dependent on a remodelled actin network (Table 4). An identical outcome was obtained when actin remodelling was attenuated by blocking Ca^2+^ plume generation with nifedipine (Tables 3, 4). These data support the conclusion that endocytosis was enhanced during WI papillae construction and that the remodelled actin network was required to polarize vesicle trafficking to the outer periclinal cytoplasm of the epidermal cells.

**Table 4.** *Effects of actin network remodelling and cytosolic Ca* ^*2+*^ *plumes on vesicle distribution in* trans-*differentiating adaxial epidermal cells of culturedV.* faba *cotyledons.* Cotyledons were freshly harvested (0 h, control), or cultured for 15 h in the absence/presence of an endo- and exocytosis inhibitor, Brefeldin A (BFA), an actin depolymerization drug, latrunculin B, an actin stabilization drug, jasplakinolide, or a DHP-receptor Ca^2+^-channel blocker, nifedipine. Thereafter, transverse sections of treated cotyledons were stained with the membrane dye FM4-64FX for 10 min. Fluorescence was measured as total pixel intensities in specified cell regions. These values were adjusted for fluorescence from FM4-64FX located in the plasma membrane using the BFA values (for more details, see Supplementary Table S1) to provide estimates of cytoplasmic fluorescence. Data are the mean differences ±SE (*n*=4) between mean pixel intensities of 25 cells measured per cotyledon recorded across four replicate cotyledons for the specified treatment minus fluorescence derived from a similar population of cells exposed to BFA. See Supplementary Table S1 for procedure used to estimate the SE of the difference between two means of unpaired observations.

Treatment	Fluorescence intensity (arbitrary units)			
	Outer periclinal cytoplasm	Anticlinal cytoplasm	Inner periclinal cytoplasm	Total
0 h Control	43 ± 7	0	33 ± 6	76 ± 10
15 h Control	385 ± 17	25 ± 5	13 ± 4	424 ± 19
15 h Latrunculin B (100 nM)	184 ± 18	121 ± 13	180 ± 15	505 ± 27
15 h Jasplakinolide (100 nM)	184 ± 25	101 ± 10	176 ± 25	461 ± 37
15 h Nifedipine (100 μM)	165 ± 11	111 ± 7	142 ± 8	418 ± 15

### Exo- and endocytosis are involved in construction of wall ingrowth papillae

To assess the influence of exo-/endocytosis on construction of WI papillae, cotyledons were first cultured for 9 h on MS medium to ensure that deposition of the uniform wall layer in their *trans*-differentiating adaxial epidermal cells was completed (Zhang *et al.*, 2015*d*). They were then subjected to a 4-h pre-treatment at 4 °C in the absence/presence of exo-/endocytosis inhibitors to ensure their penetration, and this was followed by a further 6 h of culture at 26 °C. The putative myosin inhibitor 2,3-butanedione 2-monoxime (BDM; Samaj *et al.*, 2000) was used to inhibit exocytosis and the generic endocytotic inhibitor Wortmannin (Spiro *et al.*, 1996) was used to prevent endocytosis. Estimates of vesicle trafficking using FM4-64FX were obtained at the 9 h and 9 + 6 h culture periods, as previously described.

For control cotyledons, the amount of endocytosed plasma membrane declined by 26.5% between 9 and 15 h of culture, and this was accounted for entirely by decreased fluorescence in the outer periclinal cytoplasm (Table 5). Potential inhibition of myosin-dependent exocytosis with BDM led to endocytosed vesicles being distributed equally throughout the entire cytoplasm without impacting overall endocytosis (Table 5). This suggests there is probably a considerable re-cycling of endocytosed vesicles within the outer periclinal cytoplasm. In contrast, treatment with Wortmannin predictably blocked endocytosis by 86% (Table 5). In the presence of either inhibitor, WI papillae formation was diminished (Table 5), indicating that it depends upon both exo- and endocytosis.

**Table 5.** *Effect of exo- and endocytosis on vesicle distribution and WI papillae construction in* trans*-differentiating adaxial epidermal cells of cultured* V. faba *cotyledons.* Cotyledons were cultured for 9 h to ensure deposition of the uniform wall layer (Zhang *et al.*, 2015*d*) prior to transfer to media with/without exo-/endocytosis inhibitors at 4 °C for 4 h, and thereafter for a further 6 h of culture at 26 °C. The myosin motor inhibitor 2,3-butanedione 2-monoxime (BDM) and the endocytosis inhibitor Wortmannin were used. Fluorescence of the membrane dye FM4-64FX was measured as total pixel intensities in specified cell regions, to obtain estimates of cytoplasmic fluorescence. These values were adjusted for fluorescence detected in the plasma membrane alone from measures of cotyledons cultured on Brefeldin A (BFA; for more details, see Supplementary Table S1). Data are means ±SE of pixel intensities of 25 cells measured per cotyledon recorded across four replicate cotyledons for the specified treatment minus fluorescence derived from a similar population of cells exposed to BFA (*n*=100), and means ±SE of percentage of cells with WI papillae determined from 100 cells per cotyledon and six replicate cotyledons (*n*=6).

Treatment	FM4-64FX fluorescence intensity (arbitrary units) in				% cells with WI papillae
	Outer periclinal cytoplasm	Anticlinal cytoplasm	Inner periclinal cytoplasm	Total	
9 h Control	513 ± 26	35 ± 7	0	548 ± 27	57 ± 2
9 h+6 h Control	364 ± 13	20 ± 4	19 ± 7	403 ± 15	89 ± 3
9 h+6 h BDM (50 mM)	159 ± 11	102 ± 15	174 ± 12	435 ± 22	59 ± 4
9 h+6 h Wortmannin (33 μM)	24 ± 7	0	32 ± 8	56 ± 10	64 ± 3

### Differential and epidermal TC-specific expression of key actin-binding and vesicle-trafficking genes coincide with construction of the uniform wall layer or wall ingrowth papillae

Since actin-independent vesicle trafficking accounts for polarized construction of the uniform wall layer (Fig. 2G–I; Table 1), our search focused on genes encoding proteins associated with vesicle trafficking, such as vesicle tethering, docking, or fusion to the plasma membrane (Table 6; see Supplementary Table S2 for expression details of identified genes). The exocyst functions as a vesicle-tethering complex and is comprised of eight subunits (van de Meene *et al.*, 2017) of which a *VfSEC3a/b* isoform and an EXO70 paralog, *VfEXO70H7*, were specifically up-regulated during construction of the uniform wall layer (Table 6, Supplementary Table S2). Expression of a further three exocyst components, namely another *VfSEC3a/b* isoform, *VfEXO70B2*, and *VfEXO70E1*, remained up-regulated during WI formation (Table 6, Supplementary Table S2). VfSNAREs are integral membrane proteins present in both vesicle and target membranes that interact to form a SNARE complex to effect vesicle docking and fusion (Gendre *et al.*, 2015). Expression of a SNARE member, *VfVAMP722*, met our search criteria (Table 6, Supplementary Table S2).

**Table 6.** *Transfer cell-specific differentially expressed genes (DEGs) encoding proteins involved in actin remodelling or the endomembrane secretory system during formation of the uniform wall layer and WI papillae in* trans*-differentiating adaxial epidermal cells of cultured* V. faba *cotyledons* For each DEG, gene ID, annotation, and percentage of amino acid identity of the encoded protein to the Arabidopsis (At) homolog are given. The bioinformatic data are presented in Supplementary Table S2.

DEGs during uniform wall layer formation				DEGS during WI papillae formation			
Gene ID	Annotation	At homolog ID	%	Gene ID	Annotation	At homolog ID	%
**Actin-binding proteins**							
U1054	VfFH8*	1G59910	54	U17250	*VfFH1*	3G25500	69
U9176	VfADF1*	3546010	83	CL5671.C2	VfADF3**	5G59880	79
				CL923.C2	VfVLN3**	3G57410	69
**Vesicle trafficking**							
Exocytosis							
CL4811.C4	*VfSEC3a/b*	1G47550	82	CL13784.C1U7468; 10359; 13407; 13408; 13410	*Vfmyosin* *XI-1*	4G33200	73
U21554	*VfEXO70H7*	5G58730	46	U12467	*VfAGD14*	1G08680	77
CL4811.C2	VfSEC3a/b*	1G47560	78				
U12008	VfEXO70B2*	1G07000	57				
U23669	VfEXO70E1*	3G29400	47				
U21726	*VfVAMP722*	2G33120	65				
Endocytosis							
				CL18504	*VfDRP1E*	3G6019	80

* DEGs sustained across the formation of the uniform wall layer and WI papillae are included in the uniform wall layer gene cohort.

** In specified cases, where encoded proteins are known to be Ca^2+^-sensitive, the criteria for TC-specific differentially expressed genes were relaxed as the Ca^2+^ signal is specific to the epidermal cell (Zhang *et al.*, 2015*a*).

Guided by WI papillae construction being regulated by Ca^2+^-dependent re-modelling of the actin network (Figs 2, 3, 5, 6; Tables 2, 3), we identified transcripts of three up-regulated actin-severing proteins. These were two Class 1 formins (*FORMIN-HOMOLGY1*), *VfFH1* and *VfFH8*, and an *ACTIN DEPOLYMERIZING FACTOR*, *VfADF1* (Table 6, Supplementary Table S2; Li *et al.*, 2015). Upon relaxing the search criteria and interrogating the RNAseq database for transcripts of Ca^2+^-activated actin binding proteins, we identified a constitutively expressed actin-severing *ADF*, *VfADF4* (Li *et al.*, 2015), and a group II villin, *VfVLN3* (Huang *et al.*, 2015; Table 6, Supplementary Table S2). The absence of Ca^2+^ plumes in the underlying storage parenchyma cells (Zhang *et al.*, 2015*a*) suggests that elevated actin-binding activity of their encoded proteins would be confined to *trans*-differentiating epidermal cells.

Pharmacological studies demonstrated that polarized vesicle trafficking was dependent upon a remodelled actin network (Table 4) and that both exo- and endocytosis contribute to WI papillae formation (Table 5). Polarized vesicle delivery along the remodelled actin network (Table 4) by myosin to developing WI papillae (Table 5) is consistent with the WI papillae-specific up-regulated expression of *VfmyosinX1-1* and a class 4 *ADP-ribosylation factor GTPase-activating domain* (*AGD*) *protein*, *AGD14* (Table 6, Supplementary Table S2; Ueda *et al.*, 2015). The only detected up-regulated gene encoding a protein involved in exocytosis during WI papillae construction was another *VfSEC3a/b* isoform. This was accompanied by sustained up-regulated expression of other exocyst components, *VfEXO70B2*, *VfEXO70E1*, and a *VfSEC3a/b* isoform (Table 6, Supplementary Table S2).

Endocytotic activity associated with construction of WI papillae (Table 6, Supplementary Table S2) was marked by differential regulation of a member of the plant-specific dynamin-related protein 1 subfamily, *VfDRP1E*, which is responsible for severing cargo-containing endocytotic vesicles from the plasma membrane (Paez Valencia *et al.*, 2016). To test whether the *VfDRP1E* isoform contributed to assembling WI papillae, the catalytic activities of dynamin protein super family members were inhibited by transferring 9-h cultured cotyledons to MS medium containing the dynamin inhibitors dynasore or dyngo4a (McCluskey *et al.*, 2013). Both inhibitors blocked endocytosis (Table 7). The absence of any effect by the biologically inactive analogue Dyno Φ (McCluskey *et al.*, 2013) on FM4-64FX accumulation or distribution within the epidermal cells suggests that dynasore and dyngo4a specifically inhibited DRP1E activity to form endocytotic vesicles (Table 7). The inhibition of WI papillae formation under these conditions points to DRP1E regulating endocytosis associated with construction of WI papillae.

**Table 7.** *Effect of dynamin activity on WI papillae construction and vesicle distribution in* trans-*differentiating adaxial epidermal cells of cultured* V. faba *cotyledons.* Cotyledons were cultured for 9 h to ensure deposition of the uniform wall layer (Zhang *et al.*, 2015*d*) prior to transfer to media with/without dynamin inhibitors at 4 °C for 4 h, and thereafter for a further 6 h of culture at 26 °C. The inhibitors tested were two dynamin-family inhibitors, Dynasore and Dyngo 4, and a biologically inactive Dynasore/Dyngo analogue, Dyngo Φ. Fluorescence of the membrane dye FM4-64FX was measured as total pixel intensities in specified cell regions, to obtain estimates of cytoplasmic fluorescence. These values were adjusted for fluorescence detected in the plasma membrane alone from measures of cotyledons cultured on Brefeldin A (BFA; for more details, see Supplementary Table S1). Data are means ±SE of pixel intensities of 25 cells measured per cotyledon recorded across four replicate cotyledons for the specified treatment minus fluorescence derived from a similar population of cells exposed to BFA (*n*=100), and mean ±SE of percentage of cells with WI papillae determined from 100 cells per cotyledon and six replicate cotyledons (*n*=6).

Treatment	FM4-64FX fluorescence intensity (arbitrary units)				% cells with WI papillae
	Outer periclinal cytoplasm	Anticlinal cytoplasm	Inner periclinal cytoplasm	Total	
9 h Control	520 ± 22	24 ± 7	22 ± 9	566 ± 25	58 ± 3
9 h+6 h Control	368 ± 13	28 ± 7	23 ± 6	429 ± 22	88 ± 4
9 h+6 h Dynasore (100 μM)	0	0	39 ± 7	39 ± 7	62 ± 3
9 h+6 h Dyngo 4a (20 μM)	36 ± 7	0	27 ± 6	63 ± 9	57 ± 4
9 h+6 h Dyngo Φ (20 μM)	381 ± 13	14 ± 5	25 ± 8	420 ± 16	86 ± 3

## Discussion

### A remodelled actin network is essential for the construction of wall ingrowth papillae, but not of the uniform wall layer

Prior to induction of *trans*-differentiation of adaxial epidermal cells of *V. faba* cotyledons to a TC morphology, the actin network throughout their outer periclinal cytoplasm was comprised of thick actin bundles arranged parallel to the long axis of the cell (Figs 2A, 3A, 4A, C, E, G; Table 2). Upon transfer of cotyledons to culture medium, their adaxial epidermal cells *trans*-differentiated to a TC morphology by depositing a uniform wall layer over the original outer periclinal wall on which WI papillae were assembled (Fig. 2G, J; Table 1). During *trans*-differentiation, their actin network underwent fragmentation, with the long parallel actin bundles being replaced by short, thin actin bundles (Fig. 2D; Table 2); a similar pattern of actin remodelling occurs in nematode-induced giant cells (Favery *et al.*, 2016).

Depolymerization or stabilization of the actin network (Fig. 2B, E and C, F) resulted in selective inhibition of WI papillae formation without affecting deposition of the polarized uniform wall layer (Fig. 2H, K and I, L; Table 1). Actin-independent formation of the uniform wall layer, polarized to the outer periclinal wall of adaxial epidermal cells, contrasts with actin-dependent cell wall synthesis in other polarized cell systems, including root tips, pollen tubes, and defense papillae (Hardham, 2013; Grierson *et al.*, 2014; Qu *et al.*, 2015). Perhaps the polarized delivery of vesicles carrying cell wall cargoes to construct the uniform wall layer, independent of trafficking along actin bundles, must rely on a short post-Golgi path (less than 1 µm; Zhang *et al.*, 2015*a*) combined with the strongly up-regulated expression of genes encoding proteins that control the tethering, docking, and fusion of vesicles to the outer periclinal wall plasma membrane of the adaxial epidermal cells (Table 6, Supplementary Table S2).

To our knowledge this is the first report of actin-dependent formation of WI papillae in cells developing to a TC morphology. The caveat to this claim is whether suppression of WI papillae formation in nematode-induced giant cells by actin-modifying drugs results from their direct action or indirectly from compromising giant-cell expansion (Favery *et al.*, 2016). Overall, actin dependence of the assembly of WI papillae aligns most closely with that observed for formation of wall papillae or appositions as a defense response of epidermal cells to impede hyphal penetration of pathogenic fungi (Hardham, 2013). However, there is a substantive difference in size and cellular organization of these two types of papillae. Diameters of WI papillae of TCs are an order of magnitude smaller than those of defense papillae (380 nm versus 5–10 µm; Zhang *et al.*, 2015*a* and Zeyen *et al.*, 2002, respectively). Moreover, WI papillae of TCs occur in high densities (e.g. 352, 000 WI papillae mm^−2^ of outer periclinal wall of adaxial epidermal cells). Thus, the actin array servicing each WI papilla must, of necessity, be finely organized to regulate their intrusive development into the cytoplasm.

Insights into how the remodelled actin network contributes to assembling WI papillae were deduced from examining the concurrence of the spatiotemporal dynamics of actin bundle remodelling with that of WI papillae assembly. Based on the role that actin bundles play in providing tracks for intracellular trafficking of vesicles, impacts of exo-/endocytosis on WI papillae construction are now discussed.

### Spatiotemporal dynamics of actin remodelling during *trans*-differentiation to a transfer cell morphology

**Fig. 7.**
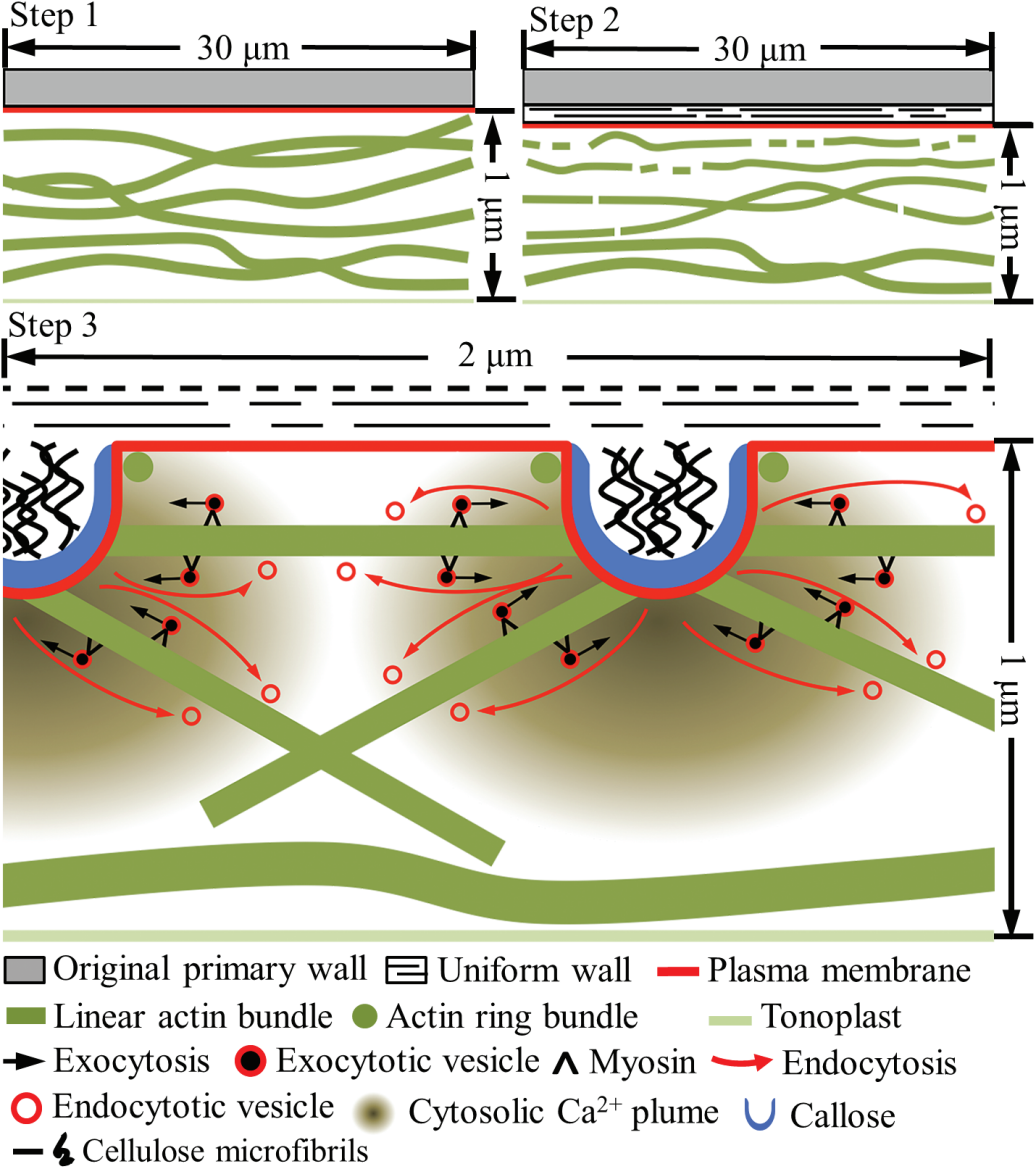
Proposed model of the spatiotemporal relationship between Ca^2+^-dependent actin network remodelling, vesicle trafficking, and WI papillae assembly in cotyledon adaxial epidermal cells *trans*- differentiating to a TC morphology. The figure panels are drawn to an approximately relative scale. Step 1: prior to induction of *trans*-differentiation, long actin bundles are aligned parallel to the longitudinal axis of the outer periclinal cell wall throughout the underlying cytoplasm. Step 2: on induction and deposition of the uniform wall layer, the actin bundles proximal to the outer periclinal cell wall/cytoplasmic interface become thinner and begin to fragment (this diagram is a composite of Fig. 2B, C). Step 3: mediated by plumes of elevated [Ca^2+^]_cyt_ that define loci at which WI papillae arise, these processes continue to ultimately produce short, thin actin bundles. Exocytotic vesicles are delivered to developing WI papillae by myosin motors travelling along the short, thin actin bundles. The bundles are oriented horizontally or vertically by [Ca^2+^]_cyt_ gradients arising respectively from the flanks or tips of developing WI papillae. Vesicles carrying cellulose and callose synthases are probably delivered to WI papillae tips while pectins and xyloglucans may be delivered to their flanks. Vesicle recycling by actin-independent endocytotic activity occurs, but its location on WI papillae is unclear.

The thick, long actin bundles, aligned parallel to the long axis of the epidermal cells, were remodelled during *trans*-differentiation to a TC morphology (Fig. 7 Step 1). The first detectable structural manifestation of bundle remodelling was a decrease in their width accompanied by the commencement of fragmentation (Figs 3B, 7 Step 2). Bundle thinning probably resulted from actin filaments being peeled off the bundles through depolymerization (Li *et al.*, 2015). As found for pollen tubes, root hairs, and nematode-induced giant cells, actin filament depolymerization was probably catalysed by an enhanced activity of VfADF1 and VfFH8 homologs, mediated by a TC-specific up-regulated expression of their encoding genes during WI papillae construction (Table 6, Supplementary Table S2; Grierson *et al.*, 2014; Qu *et al.*, 2015; Favery *et al.*, 2016). Subsequent thinning and severance of the long, thinner bundles into shorter fragments (Table 2; Fig. 3C, D) closely followed the temporal profile of cells forming WI papillae (Fig. 3E).

That actin remodelling is an essential precursor for WI papillae construction (Fig. 3E) is further suggested by the shared regulation of actin bundle severance and WI papillae assembly by a TC-specific rise in [Ca^2+^]_cyt_ levels organized into inward-directed plumes (Table 3; Figs 6, 7 Step 3; Zhang *et al.*, 2015*a*, 2015*c*). This finding is consistent with [Ca^2+^]_cyt_ gradients orchestrating the longitudinal organization of actin filament/bundles in root hairs and pollen tubes to drive polarized tip growth (Grierson *et al.*, 2014; Qu *et al.*, 2015). A comparable spatial relationship exists in the epidermal TCs. Here, a declining inward-directed gradient of [Ca^2+^]_cyt_ from the outer periclinal cell wall/cytoplasmic interface (Zhang *et al.*, 2015*a*) corresponded with an increase in longer and thicker actin bundles. Indeed, by 750 nm from the interface, the bundle dimensions and organization were identical to those in epidermal cells before undergoing *trans*-differentiation (Table 2; Figs 4, 7 Step 3).

The strong regulation by [Ca^2+^]_cyt_ levels on actin remodelling points to Ca^2+^-activated actin-depolymerizing enzymes exerting predominant control over actin organization in the epidermal TCs. Candidates for these enzymes were the constitutively expressed *VfADF3* (Inada, 2017) and a group II villin, *VfVLN3*, that, depending upon [Ca^2+^]_cyt_, can function to either sever (high [Ca^2+^]_cyt_) or bundle (low [Ca^2+^]_cyt_) actin filaments (Table 6, Supplementary Table S2; Huang *et al.*, 2015). Whether depolymerization and severance of pre-existing actin filaments is the sole source for the turnover of actin bundles is uncertain (Li *et al.*, 2015). In this context, the product of up-regulated expression of *VfFH1* (Table 6, Supplementary Table S2) could initiate bundle formation through the known capacity of FH1to nucleate actin filaments, as found for nematode-induced giant cells and pollen tubes (Favery *et al.*, 2016; Qu *et al.*, 2016). At the same time, VfFH1 could also act as an anchor to bridge fragmented actin bundles to the cell wall (Martinière *et al.*, 2011) and hence help to determine the loci of deposition of WI papillae.

Lengths and widths of the shortest actin bundles in the epidermal TCs were two to four times less than those forming the actin fringe of pollen tubes (Table 2; e.g. Chang and Huang, 2015; Qu *et al.*, 2015). Their shorter lengths could be related to delivering vesicles/organelles to developing WI papillae. Here, horizontally oriented actin bundles would need to fit between adjoining papillae with diameters of 380 nm and spaced at 1.5 µm centre-to-centre (Zhang *et al.*, 2015*a*). This distance of 1.1 µm corresponds to the lengths of the short actin bundles interfacing with (Table 2; Fig. 5D), and possibly anchored by FH1 to, WI papillae (as proposed in Fig. 7, Step 3; Table 6, Supplementary Table S2; van de Meene, 2017). Moreover, their horizontal orientation corresponds with the gradient in [Ca^2+^]_cyt_ located between adjoining Ca^2+^ plumes (Zhang *et al.*, 2015*a*). Similarly, the vertically oriented actin bundles align with the inward-directed [Ca^2+^]_cyt_ gradient that emanates from the tips of developing WI papillae (Fig. 7 Step 3; Zhang *et al.*, 2015*a*). Interestingly, the circular-shaped actin bundles, most proximal to the outer periclinal cell wall/cytoplasmic interface, were organized into rings (Figs 5A, 7). Their diameters and distribution patterns suggested that they could enclose each WI papillae (Figs 5A, 7;Table 2). Ring-like actin bundles, termed aquosomes, have been detected in root hairs and pollen tubes (Smertenko *et al.*, 2010; Vogler and Sprunck, 2015). While the function of aquosomes has not been elucidated, we speculate that those apparently ensheathing the bases of WI papillae could delineate loci for their assembly.

### Vesicle trafficking and assembly of wall ingrowth papillae

Extension of WI papillae from their sites of initiation on the uniform wall layer into the epidermal cytoplasm probably occurs as a progressive accretion of cell wall material deposited at their tips, as evidenced by their cylindrical shape and inner core of whorled cellulose microfibrils orientated perpendicular to the uniform wall layer, embedded in a matrix of polysaccharides and enclosed by a callose sheath (Fig. 7 Step 3; Talbot *et al.*, 2007; Vaughn *et al.*, 2007). This form of development must rely on a finely tuned delivery of secretory vesicles first to multiple discrete loci on the uniform wall layer at which WI papillae are initiated, and thereafter to their tips for further development.

Labelling endocytotic vesicles with the fluorescent styryl dye FM4-64FX (Bolte *et al.*, 2004) demonstrated that the TC vesicle population was preferentially localized to their outer periclinal cytoplasm. Similar to tip-growth systems, polarized distribution of vesicles was dependent upon a [Ca^2+^]_cyt_-regulated remodelling of the actin network that delivered cargos essential for WI papillae assembly (Tables 4, 5; Gu and Nielsen, 2013). The combined dissipation of polarized vesicle distribution and inhibition of WI papillae assembly by the myosin inhibitor BDM points to vesicles and/or organelles being trafficked along the actin bundles by myosin motor proteins (Fig. 7 Step 3; Table 5; Samaj *et al.*, 2000). Significantly, a myosin X1 paralog, *Vf myosin XI-1*, was strongly up-regulated during WI papillae formation (Table 6, Supplementary Table S2). Interestingly, among the 13 members of the Arabidopsis (At) class XI myosins, At myosin XI-1 is phylogenetically distant, and biochemically distinct, from the other 12 paralogs (Peremyslov *et al.*, 2012; Haraguchi *et al.*, 2016). The biochemical properties of myosin XI-1 suggest an exclusive function as a regulator of organelle movement/tethering (Haraguchi *et al.*, 2016). The TC-specific up-regulated expression of the gene encoding the membrane-localized ADP-ribosylation factor GTPase-activating domain (AGD) protein, *VfAGD14* homolog (Table 6, Supplementary Table S2), could be responsible for vesicle trafficking to specific membrane domains located at the tips of the WI papillae (Vernoud *et al.*, 2003; Yoo *et al.*, 2012). Collectively, these functions fit nicely with the ubiquitous aggregation of organelles, including Golgi, around developing WI papillae (Offler *et al.*, 2003); an organization that is consistent with the intimate spatial relationship between the short actin bundles and developing WI papillae (Fig. 5). As a consequence, post-Golgi vesicle transport to WI tips is exceptionally short compared with vesicle flows in the clear zone of pollen tubes (Helper and Winship, 2015). Tethering and fusion of the trafficked vesicles to the targeted plasma membrane domains of WI papillae could be mediated by up-regulated components of the exocyst complex (i.e. VfEXO70E1 and VfEXO70B2; Table 6, Supplementary Table S2) that participates in polarized, spatiotemporal secretion phenomena (van de Meene *et al.*, 2017).

The five-fold increase in endocytotic activity during WI papillae formation, localized to the outer-periclinal cytoplasm (Table 4), is consistent with a large membrane surface-area to volume ratio of the vesicles fusing with the pre-existing plasma membrane covering the apical poles of WI papillae, thus requiring retrieval of the excess plasma membrane. Following a similar approach to Ketelaar *et al.* (2008), the extent of retrieval of plasma membrane by endocytosis during construction of a WI papilla was estimated to be 61% of the fused membrane (see Supplementary Computational Information S1). Because of the substantial contribution to the total volume of a WI papilla by callose synthesized at the plasma membrane , the extent of retrieval by endocytosis during WI papillae formation is less than that determined for root hairs (87%) and pollen tubes (79%; Ketelaar *et al.*, 2008).

Endocytotic activity in the *trans*-differentiating epidermal cells appeared to be actin independent (Fig. 7 Step 3; Table 4). This equates with findings for endocytosis occurring at the tips, but not shanks, of elongating pollen tubes (Hepler and Winship, 2015). The increased endocytotic activity during WI papillae formation coincided with the epidermal TC-specific up-regulated expression of the dynamin related protein (DRP) homolog, *VfDRP1E* (Table 6, Supplementary Table S2). In broad terms, dynamins are a large family of GTPase proteins that mediate tubulation and scission of the endocytosed membrane (Fujimoto and Tsutsumi, 2014). DRP1E belongs to the DRP1 sub-family, which is comprised of five members (DRP1A–E) in Arabidopsis. Interestingly, AtDRP1E accumulates in sphingolipid and sterol-enriched plasma membrane microdomains in cold-acclimated Arabidopsis (Minami *et al.*, 2015), possibly indicating the operation of a microdomain-associated endocytotic pathway (Fan *et al.*, 2015). Consistent with a central role played by endocytosis in WI papillae construction was the finding that the endocytotic-specific blockers Wortmannin (Wang *et al.*, 2013) and dynasore analogs (McCluskey *et al.*, 2013) inhibited WI papillae assembly (Tables 4, 7). This points to the bulk of endocytosed vesicles being recycled into the secretory pathway of the *trans*-differentiating epidermal cells (Fig. 7, Step 3; Table 4), as found for elongating pollen tubes (Wang *et al.*, 2013).

Overall, this study demonstrates that assembly of WI papillae, but not the polarized uniform wall layer, is dependent upon exocytotic vesicle trafficking along a finely organized Ca^2+^-mediated remodelled actin network. In contrast, the significant retrieval of fused membrane by endocytosis is actin independent. Significant unanswered issues that require clarification in future studies include the spatial relationship between exo- and endocytosis and how exocytosed pectins and hemicelluloses move across the callose sheath to reach the inner cellulose core of WI papillae.

## Supplementary data

Supplementary data are available at *JXB* online.

Table S1. Effects of actin network remodelling and cytosolic Ca^2+^ plumes on vesicle distribution.

Table S2. Key transcripts encoding proteins involved in actin remodelling or the endomembrane secretory system.

Table S3. Key transcripts reported in Zhang *et al.* (2015*d*) that do not satisfy the criteria used to select encoding genes in Table S2.

Table S4. Statistical verification of spatial association between actin bundles and WI papillae (including coding for the R software used).

Computational Information S1. Estimation of portion of exocytosed plasma membrane retrieved by endocytosis.

## Supplementary Material

Supplementary_Tables_S1_S4

## Abbreviations:

AVG: Aminoethoxyvinylglycine
BDM: 2,3-butanedione 2-monoxime
CLSM: confocal laser scanning microscope
DHP: dihydropyridine
DRP: dynamin related protein
EGTA: ethylene glycol tetraacetic acid
FDR: false discovery rate
MS: Murashige and Skoog
PBS: phosphate-buffered saline
PIPES: piperazine-N,N-bis(2-ethanesulfonic acid)
ROS: reactive oxygen species
RPKM: mapped reads per kilo base per million reads
TC: transfer cell
UWL: uniform wall layer
WI: wall ingrowth.

## References

[CIT0001] Andriunas FA, Zhang HM, Xia X, Offler CE, McCurdy DW, Patrick JW 2012 Reactive oxygen species form part of a regulatory pathway initiating *trans*-differentiation of epidermal transfer cells in *Vicia faba* cotyledons. Journal of Experimental Botany 63, 3617–3629. 22442421 10.1093/jxb/ers029 PMC3388844

[CIT0002] Andriunas FA, Zhang HM, Xia X, Patrick JW, Offler CE 2013 Intersection of transfer cells with phloem biology—broad evolutionary trends, function, and induction. Frontiers in Plant Science 4, 221. 23847631 10.3389/fpls.2013.00221 PMC3696738

[CIT0003] Baddeley A, Rubak E, Turner R 2015 Spatial point patterns: methodology and applications with R. London: Chapman and Hall/CRC Press.

[CIT0004] Bolte S, Talbot C, Boutte Y, Catrice O, Read ND, Satiat-Jeunemaitre B 2004 FM-dyes as experimental probes for dissecting vesicle trafficking in living plant cells. Journal of Microscopy 214, 159–173. 15102063 10.1111/j.0022-2720.2004.01348.x

[CIT0005] Cárdenas L, Lovy-Wheeler A, Wilsen KL, Hepler PK 2005 Actin polymerization promotes the reversal of streaming in the apex of pollen tubes. Cell Motility and the Cytoskeleton 61, 112–127. 15849722 10.1002/cm.20068

[CIT0006] Chang M, Huang S 2015 Arabidopsis ACT11 modifies actin turnover to promote pollen germination and maintain the normal rate of tube growth. The Plant Journal 83, 515–527. 26096143 10.1111/tpj.12910

[CIT0007] Dibley SJ, Zhou Y, Andriunas FA, Talbot MJ, Offler CE, Patrick JW, McCurdy DW 2009 Early gene expression programs accompanying *trans*-differentiation of epidermal cells of *Vicia faba* cotyledons into transfer cells. New Phytologist 182, 863–877. 19383101 10.1111/j.1469-8137.2009.02822.x

[CIT0008] Fan L, Li R, Pan J, Ding Z, Lin J 2015 Endocytosis and its regulation in plants. Trends in Plant Science 20, 388–397. 25914086 10.1016/j.tplants.2015.03.014

[CIT0009] Farley SJ, Patrick JW, Offler CE 2000 Functional transfer cells differentiate in cultured cotyledons of *Vicia faba* seeds. Protoplasma 214, 102–117.

[CIT0010] Favery B, Quentin M, Jaubert-Possamai S, Abad P 2016 Gall-forming root-knot nematodes hijack key plant cellular functions to induce multinucleate and hypertrophied feeding cells. Journal of Insect Physiology 84, 60–69. 26211599 10.1016/j.jinsphys.2015.07.013

[CIT0011] Fujimoto M, Tsutsumi N 2014 Dynamin-related proteins in plant post-Golgi traffic. Frontiers in Plant Science 5, 408. 25237312 10.3389/fpls.2014.00408 PMC4154393

[CIT0012] Gendre D, Jonsson K, Boutté Y, Bhalerao RP 2015 Journey to the cell surface–the central role of the trans-Golgi network in plants. Protoplasma 252, 385–398. 25187082 10.1007/s00709-014-0693-1

[CIT0013] Grierson C, Nielsen E, Ketelaarc T, Schiefelbein J 2014 Root hairs. The Arabidopsis Book 12, e0172. 24982600 10.1199/tab.0172 PMC4075452

[CIT0014] Gu F, Nielsen E 2013 Targeting and regulation of cell wall synthesis during tip growth in plants. Journal of Integrative Plant Biology 55, 835–846. 23758901 10.1111/jipb.12077

[CIT0015] Haraguchi T, Tominaga M, Nakano A, Yamamoto K, Ito K 2016 Myosin XI-I is mechanically and enzymatically unique among class-XI myosins in Arabidopsis. Plant & Cell Physiology 57, 1732–1743. 27273580 10.1093/pcp/pcw097

[CIT0016] Hardham AR 2013 Microtubules and biotic interactions. The Plant Journal 75, 278–289. 23480445 10.1111/tpj.12171

[CIT0017] Hepler PK, Winship LJ 2015 The pollen tube clear zone: clues to the mechanism of polarized growth. Journal of Integrative Plant Biology 57, 79–92. 25431342 10.1111/jipb.12315

[CIT0018] Holzinger A, Meindl U 1997 Jasplakinolide, a novel actin targeting peptide, inhibits cell growth and induces actin filament polymerization in the green alga *Micrasterias*. Cell Motility and the Cytoskeleton 38, 365–372. 9415378 10.1002/(SICI)1097-0169(1997)38:4<365::AID-CM6>3.0.CO;2-2

[CIT0019] Huang S, Qu X, Zhang R 2015 Plant villins: versatile actin regulatory proteins. Journal of Integrative Plant Biology 57, 40–49. 25294278 10.1111/jipb.12293

[CIT0020] Inada N 2017 Plant actin depolymerizing factor: actin microfilament disassembly and more. Journal of Plant Research 130, 227–238. 28044231 10.1007/s10265-016-0899-8 PMC5897475

[CIT0021] Ketelaar T, Galway ME, Mulder BM, Emons AM 2008 Rates of exocytosis and endocytosis in Arabidopsis root hairs and pollen tubes. Journal of Microscopy 231, 265–273. 18778424 10.1111/j.1365-2818.2008.02031.x

[CIT0022] Li J, Blanchoin L, Staiger CJ 2015 Signaling to actin stochastic dynamics. Annual Review of Plant Biology 66, 415–440. 10.1146/annurev-arplant-050213-040327 25423079

[CIT0023] Li X, Li JH, Wang W 2014 ARP2/3 complex-mediated actin dynamics is required for hydrogen peroxide-induced stomatal closure in Arabidopsis. Plant, Cell & Environment 37, 1548–1560. 10.1111/pce.12259 24372484

[CIT0024] Lohse M, Nagel A, Herter T, May P, Schroda M, Zrenner R, Tohge T, Fernie AR, Stitt M, Usadel B 2014 Mercator: a fast and simple web server for genome scale functional annotation of plant sequence data. Plant, Cell & Environment 37, 1250–1258. 10.1111/pce.12231 24237261

[CIT0025] Martinière A, Gayral P, Hawes C, Runions J 2011 Building bridges: formin1 of Arabidopsis forms a connection between the cell wall and the actin cytoskeleton. The Plant Journal 66, 354–365. 21241388 10.1111/j.1365-313X.2011.04497.x

[CIT0026] McCluskey A, Daniel JA, Hadzic G 2013 Building a better dynasore: the dyngo compounds potently inhibit dynamin and endocytosis. Traffic 14, 1272–1289. 24025110 10.1111/tra.12119 PMC4138991

[CIT0027] Minami A, Tominaga Y, Furuto A, Kondo M, Kawamura Y, Uemura M 2015 Arabidopsis dynamin-related protein 1E in sphingolipid-enriched plasma membrane domains is associated with the development of freezing tolerance. The Plant Journal 83, 501–514. 26095877 10.1111/tpj.12907

[CIT0028] Murashige T, Skoog F 1962 A revised medium for rapid growth and bioassays with tobacco tissue cultures. Physiologia Plantarum 15, 473–497.

[CIT0029] Offler CE, Liet E, Sutton EG 1997 Transfer cell induction in cotyledons of *Vicia faba L*. Protoplasma 200, 51–64.

[CIT0030] Offler CE, McCurdy DW, Patrick JW, Talbot MJ 2003 Transfer cells: cells specialized for a special purpose. Annual Review of Plant Biology 54, 431–454. 10.1146/annurev.arplant.54.031902.134812 14502998

[CIT0031] Paez Valencia J, Goodman K, Otegui MS 2016 Endocytosis and endosomal trafficking in plants. Annual Review of Plant Biology 67, 309–335. 10.1146/annurev-arplant-043015-112242 27128466

[CIT0032] Peremyslov VV, Klocko AL, Fowler JE, Dolja VV 2012 Arabidopsis myosin XI-K localizes to the motile endomembrane vesicles associated with F-actin. Frontiers in Plant Science 3, 184. 22969781 10.3389/fpls.2012.00184 PMC3432474

[CIT0033] Qiao F, Chang XL, Nick P 2010 The cytoskeleton enhances gene expression in the response to the Harpin elicitor in grapevine. Journal of Experimental Botany 61, 4021–4031. 20675535 10.1093/jxb/erq221 PMC2935876

[CIT0034] Qu X, Jiang Y, Chang M, Liu X, Zhang R, Huang S 2015 Organization and regulation of the actin cytoskeleton in the pollen tube. Frontiers in Plant Science 5, 786. 25620974 10.3389/fpls.2014.00786 PMC4287052

[CIT0035] R Core Team 2016 R: A language and environment for statistical computing Vienna, Austria: R Foundation for Statistical Computing, https://www.R-project.org/.

[CIT0036] Ritchie ME, Phipson B, Wu D, Hu Y, Law CW, Shi W, Smyth GK 2015 *limma* powers differential expression analyses for RNA-sequencing and microarray studies. Nucleic Acids Research 43, e47. 25605792 10.1093/nar/gkv007 PMC4402510

[CIT0037] Samaj J, Peters M, Volkmann D, Baluska F 2000 Effects of myosin ATPase inhibitor 2,3-butanedione 2-monoxime on distributions of myosins, F-actin, microtubules, and cortical endoplasmic reticulum in maize root apices. Plant & Cell Physiology 41, 571–582. 10929940 10.1093/pcp/41.5.571

[CIT0038] Smertenko AP, Deeks MJ, Hussey PJ 2010 Strategies of actin reorganisation in plant cells. Journal of Cell Science 123, 3019–3028. 20699356 10.1242/jcs.071126

[CIT0039] Spector I, Shochet NR, Kashman Y, Groweiss A 1983 Latrunculins: novel marine toxins that disrupt microfilament organization in cultured cells. Science 219, 493–495. 6681676 10.1126/science.6681676

[CIT0040] Spiro DJ, Boll W, Kirchhausen T, Wessling-Resnick M 1996 Wortmannin alters the transferrin receptor endocytic pathway *in vivo* and *in vitro*. Molecular Biology of the Cell 7, 355–367. 8868465 10.1091/mbc.7.3.355 PMC275889

[CIT0041] Szymanski DB, Cosgrove DJ 2009 Dynamic coordination of cytoskeletal and cell wall systems during plant cell morphogenesis. Current Biology 19, R800–R811. 19906582 10.1016/j.cub.2009.07.056

[CIT0042] Talbot MJ, Franceschi VR, McCurdy DW, Offler CE 2001 Wall ingrowth architecture in epidermal transfer cells of *Vicia faba* cotyledons. Protoplasma 215, 191–203. 11732058 10.1007/BF01280314

[CIT0043] Talbot MJ, Wasteneys GO, Offler CE, McCurdy DW 2007 Cellulose synthesis is required for deposition of reticulate wall ingrowths in transfer cells. Plant & Cell Physiology 48, 147–158. 17169922 10.1093/pcp/pcl046

[CIT0044] Ueda H, Tamura K, Hara-Nishimura I 2015 Functions of plant-specific myosin XI: from intracellular motility to plant postures. Current Opinion in Plant Biology 28, 30–38. 26432645 10.1016/j.pbi.2015.08.006

[CIT0045] van de Meene AML, Doblin MS, Bacic A 2017 The plant secretory pathway seen through the lens of the cell wall. Protoplasma 254, 75–94. 26993347 10.1007/s00709-016-0952-4

[CIT0046] Vaughn KC, Talbot MJ, Offler CE, McCurdy DW 2007 Wall ingrowths in epidermal transfer cells of *Vicia faba* cotyledons are modified primary walls marked by localized accumulations of arabinogalactan proteins. Plant & Cell Physiology 48, 159–168. 17169921 10.1093/pcp/pcl047

[CIT0047] Vernoud V, Horton AC, Yang Z, Nielsen E 2003 Analysis of the small GTPase gene superfamily of Arabidopsis. Plant Physiology 131, 1191–1208. 12644670 10.1104/pp.013052 PMC166880

[CIT0048] Vogler F, Sprunck S 2015 F-actin forms mobile and unwinding ring-shaped structures in germinating Arabidopsis pollen expressing Lifeact. Plant Signaling & Behavior 10, e1075684. 26337326 10.1080/15592324.2015.1075684 PMC4883927

[CIT0049] Wang H, Zhuang X, Cai Y, Cheung AY, Jiang L 2013 Apical F-actin-regulated exocytic targeting of NtPPME1 is essential for construction and rigidity of the pollen tube cell wall. The Plant Journal 76, 367–379. 23906068 10.1111/tpj.12300

[CIT0050] Wardini T, Wang XD, Offler CE, Patrick JW 2007 Induction of wall ingrowths of transfer cells occurs rapidly and depends upon gene expression in cotyledons of developing *Vicia faba* seeds. Protoplasma 231, 15–23. 17602275 10.1007/s00709-007-0244-0

[CIT0051] Wilkins KA, Bancroft J, Bosch M, Ings J, Smirnoff N, Franklin-Tong VE 2011 Reactive oxygen species and nitric oxide mediate actin reorganization and programmed cell death in the self-incompatibility response of *Papaver*. Plant Physiology 156, 404–416. 21386034 10.1104/pp.110.167510 PMC3091060

[CIT0052] Xia X, Zhang HM, Andriunas FA, Offler CE, Patrick JW 2012 Extracellular hydrogen peroxide, produced through a respiratory burst oxidase/superoxide dismutase pathway, directs ingrowth wall formation in epidermal transfer cells of *Vicia faba* cotyledons. Plant Signaling & Behavior 7, 1125–1128. 22899058 10.4161/psb.21320 PMC3489643

[CIT0053] Yoo CM, Quan L, Cannon AE, Wen J, Blancaflor EB 2012 AGD1, a class 1 ARF-GAP, acts in common signaling pathways with phosphoinositide metabolism and the actin cytoskeleton in controlling Arabidopsis root hair polarity. The Plant Journal 69, 1064–1076. 22098134 10.1111/j.1365-313X.2011.04856.x

[CIT0054] Zeyen RJ, Carver TLW, Lyngkjaer MF 2002 Epidermal cell papillae. In: Bélanger RR, Bushell WR, Dik AJ, Carver TWL, eds. Powdery mildews; a comprehensive treatise. St Paul, MN, USA: APS Press, 107–125.

[CIT0055] Zhang HM, Imtiaz MS, Laver DR, McCurdy DW, Offler CE, van Helden DF, Patrick JW 2015a Polarized and persistent Ca²⁺ plumes define loci for formation of wall ingrowth papillae in transfer cells. Journal of Experimental Botany 66, 1179–1190. 25504137 10.1093/jxb/eru460 PMC4339585

[CIT0056] Zhang HM, Talbot MJ, McCurdy DW, Patrick JW, Offler CE 2015b Calcium-dependent depletion zones in the cortical microtubule array coincide with sites of, but do not regulate, wall ingrowth papillae deposition in epidermal transfer cells. Journal of Experimental Botany 66, 6021–6033. 26136268 10.1093/jxb/erv317 PMC4566988

[CIT0057] Zhang HM, van Helden DF, McCurdy DW, Offler CE, Patrick JW 2015c Plasma membrane Ca^2+^-permeable channels are differentially regulated by ethylene and hydrogen peroxide to generate persistent plumes of elevated cytosolic Ca^2+^ during transfer cell *trans*-differentiation. Plant & Cell Physiology 56, 1711–1720. 26139237 10.1093/pcp/pcv100

[CIT0058] Zhang HM, Wheeler S, Xia X, Radchuk R, Weber H, Offler CE, Patrick JW 2015d Differential transcriptional networks associated with key phases of ingrowth wall construction in *trans*-differentiating epidermal transfer cells of *Vicia faba* cotyledons. BMC Plant Biology 15, 103. 25887034 10.1186/s12870-015-0486-5 PMC4437447

[CIT0059] Zhou Y, Andriunas F, Offler CE, McCurdy DW, Patrick JW 2010 An epidermal-specific ethylene signal cascade regulates *trans*-differentiation of transfer cells in *Vicia faba* cotyledons. New Phytologist 185, 931–943. 20085619 10.1111/j.1469-8137.2009.03136.x

