# Supplementary material for: A Ca2+-dependent remodelled actin network directs vesicle trafficking to build wall ingrowth papillae in transfer cells
Source: J Exp Bot. 2017 Sep 19;68(17):4749–64. doi: 10.1093/jxb/erx315 (PMC5853249; doi:10.1093/jxb/erx315)
Supplement: Supplementary_Tables_S1_S4 [file erx315_suppl_supplementary_tables_s1_s4.pdf]

Supplementary Data – Tables and Computational Information

A Ca^{2+}-Dependent Remodelled Actin Network Directs Vesicle Trafficking to Build Wall Ingrowth Papillae in Transfer Cells

Hui-ming Zhang, Kim Colyvas, John W. Patrick and Christina E. Offler

Table S1. *Effects of actin network remodelling and cytosolic Ca^{2+} plumes on vesicle distribution in* trans-*differentiating adaxial epidermal cells of cultured V. faba cotyledons.*

Cotyledons were freshly harvested (0-h control), or cultured for 15 h in liquid MS medium in the absence/presence of a potent inhibitor of endo- and exocytosis, Brefeldin A (BFA), an actin depolymerization drug, Latrunculin B, an actin stabilization drug, jasplakinolide, or a DHP-receptor Ca^{2+} channel blocker, nifedipine. Thereafter, transverse sections of cotyledons were stained with FM4-64FX, a membrane dye, for 10 min. Fluorescence was measured as total pixel intensities in specified cell regions. Fluorescence intensities in BFA-treated cells were measured to obtain fluorescence intensities of FM4-64FX in the plasma membrane alone as BFA blocks endocytosis. Within each cytoplasmic region examined, differences in fluorescence intensities recorded in the BFA compared to those of the remaining treatments were evaluated by an unpaired, two tailed t-test using SPSS20.0 software. Statistically significant difference from the mean fluorescence intensity of the respective BFA treatment with $^*P < 0.05$ or $^{**} P < 0.01$ (mean ± SE of pixel intensities measured in 25 cells per cotyledon replicated across four replicate cotyledons; $n = 4$). Where statistical differences were found, corresponding fluorescence intensities of the BFA treatment were subtracted from those of the remaining treatments to obtain estimates of fluorescence levels in the specified cytoplasmic regions. Estimate of the SEs of the differences between means were derived by equation: $SE = SQRT\{[n(n-1)(SE_1SQD + SE_2SQD)]/[n(2n-1)]\}$.

Treatment	Fluorescence intensity (arbitrary units) in:		
	Outer periclinal region	Anticlinal region	Inner periclinal region
T = 0	186 ± 6*	123 ± 7	182 ± 8*
15 h control	528 ± 23**	130 ± 5*	163 ± 3*
15 h Brefeldin A (357 µM)	143 ± 8	105 ± 5	150 ± 5
15 h Latrunculin B (100 nM)	327 ± 25**	226 ± 18**	329 ± 20**
15 h Jasplakinolide (100 nM)	327 ± 34**	206 ± 14**	326 ± 35**
15 h Nifedipine (100 µM)	308 ± 13**	216 ± 9**	292 ± 10**

Table S2. *Annotation information and expression profiles of key transcripts encoding proteins involved in actin remodelling or the endomembrane secretory system during uniform wall layer (UWL) and wall ingrowth papillae (WI) formation in* trans-*differentiating adaxial epidermal and storage parenchyma cells of cultured* V. faba *cotyledons*.

Unless specified otherwise, reported transcripts are TC-specific differentially expressed genes (DEGs) that meet the following criteria: Number of uniquely mapped reads per kilo base per million reads (RPKM) of transcript in adaxial epidermal cells at 3 h or 12 h > 1; 2 fold change (Log_2 FC) of > 1 with a false discovery rate (FDR) corrected P value < 0.05 calculated using LimmaR (Ritchie et al., 2015) between either 0 to 3 h (UWL specific DEGs), 3 to 12 h (WI specific DEGs) or sustained up-regulation from 0 to 3 h to 12 h of cotyledon culture. In specified cases (special DEGs), where encoded proteins were known to be Ca^{2+}-sensitive, these criteria were relaxed. Function of the encoded protein was inferred by the best-fit percentage amino acid alignment with the closest Arabidopsis homolog (% identity in amino acid sequence is provided in parentheses). When multiple fragments of the same protein were identified, expression profile of each fragment was provided separately. RPKM values are means ± SE of 6 replicate batches of cotyledons in adaxial epidermal cells and 3 replicates in storage parenchyma cells.

Sequence ID	Annotation	Relative transcript level (RPKM) in:						Log_2 fold change during:			
		Adaxial epidermal cells at:			Storage parenchyma cells at:			Adaxial epidermal cells		Storage parenchyma cells	
		0 h	3 h	12 h	0 h	3 h	12 h	0 to 3 h	3 to 12 h	0 to 3 h	3 to 12 h
**UWL specific DEGs**											
**Vesicle trafficking**											
Exocytosis proteins											
U21554	*VfEXO70H7*	0.2 ± 0.0	1.3 ± 0.1	0.2 ± 0.0	0.1 ± 0.0	0.4 ± 0.0	0.2 ± 0.1	2.7*	-2.9*	1.9	-1.2
CL4811.C4	*VfSEC3a/b*	1.0 ± 0.2	8.5 ± 1.2	1.6 ± 0.2	0.3 ± 0.1	1.1 ± 0.3	0.9 ± 0.1	3.1*	-2.5*	1.9	-0.3
U21726	*VfVAMP722*	0.0 ± 0.0	17.5 ± 3.0	0.8 ± 0.3	0.0 ± 0.0	0.0 ± 0.0	0.0 ± 0.0	10.0*	-4.4*	0.0	0.0
**TC specific DEGs up-regulated during UWL deposition and sustained during WI construction**											
**Actin binding proteins**											
Formins											
U1054	*VfFH8*	0.0 ± 0.0	11.4 ± 2.3	7.9 ± 1.3	0.0 ± 0.0	0.0 ± 0.0	0.0 ± 0.0	9.2*	-0.5	0.0	0.0
Actin depolymerising proteins											
U9176	*VfADF1*	12.9 ± 1.0	27.6 ± 2.6	22.4 ± 2.6	4.6 ± 0.9	9.4 ± 0.4	4.5 ± 1.5	1.1	-0.3	1.0	-1.1
**Vesicle trafficking**											
Exocytosis proteins											
U12008	*VfEXO70B2*	0.0 ± 0.0	1.3 ± 0.4	1.6 ± 0.6	0.0 ± 0.0	0.0 ± 0.0	0.0 ± 0.0	10.0*	0.3	0.0	0.0
U23669	*VfEXO70E1*	6.4 ± 0.4	28.1 ± 2.7	18.1 ± 2.4	2.6 ± 0.2	5.9 ± 0.7	5.5 ± 1.1	2.1*	-0.6	1.2	-0.1
CL4811.C2	*VfSec3a/b*	0.3 ± 0.1	1.1 ± 0.2	3.3 ± 0.2	0.0 ± 0.0	0.0 ± 0.0	0.3 ± 0.0	2.0*	1.5*	0.0	10.0

Sequence ID	Name	Relative transcript level (RPKM) in:						Log$_2$ fold change during:			
		Adaxial epidermal cells at:			Storage parenchyma cells at:			Adaxial epidermal cells		Storage parenchyma cells	
		0 h	3 h	12 h	0 h	3 h	12 h	0 to 3 h	3 to 12 h	0 to 3 h	3 to 12 h
**WI specific DEGs**											
**Actin binding proteins**											
Formins											
U17250	*VfFH1*	12.8 ± 1.3	18.1 ± 1.4	49.1 ± 4.3	10.2 ± 1.4	21.9 ± 3.5	11.7 ± 1.3	0.5	1.4*	1.1	-0.9
**Vesicle trafficking**											
Myosin											
Cl3784.C1, U7468, U10359, U13407, U13408, U13410	*Vf(myosin) XI-1*	2.2 ± 0.3	1.4 ± 0.2	4.4 ± 0.3	2.1 ± 0.5	3.2 ± 0.8	4.2 ± 0.5	-0.7	1.6*	0.6	0.4
ADP-ribosylation factor GTPase-activating protein											
U12467	*VfAGD14*	0.4 ± 0.1	0.6 ± 0.2	2.5 ± 0.5	0.4 ± 0.2	0.7 ± 0.1	0.5 ± 0.1	0.6	2.1*	1.0	-0.6
Encodytosis proteins											
Cl8504.C1	*VfDRP1E*	2.6 ± 0.6	1.7 ± 0.4	12.8 ± 1.8	2.1 ± 0.5	3.9 ± 1.0	6.9 ± 2.4	-0.6	2.9*	0.9	0.8
**Special DEGs encoding proteins with known Ca^{2+} dependence & strongly expressed in TCs**											
**Actin binding proteins**											
Actin depolymerising proteins											
CL5671.C2	*VfADF3*	39.6 ± 5.8	48.7 ± 1.6	51.6 ± 1.6	23.0 ± 2.3	32.0 ± 4.1	21.3 ± 1.4	0.3	0.1	0.5	-0.6
Villins											
CL923.C2	*VfVLN3*	5.2 ± 0.2	8.3 ± 0.3	5.8 ± 0.3	3.1 ± 0.1	7.8 ± 0.5	7.1 ± 0.9	0.7	-0.5	1.3	-0.1

Table S3. *Annotation information and expression profiles of key transcripts encoding proteins involved in actin remodeling or the endomembrane secretory system during ingrowth wall formation in* trans-*differentiating adaxial epidermal and storage parenchyma cells of cultured* V. faba *cotyledons reported in Zhang et al. (2015d) that do not satisfy the criteria used for selecting encoding genes listed in Table S2.*

The criteria used to identify transcripts reported in Zhang et al. (2015d) were: Number of uniquely mapped reads per kilo base per million reads (RPKM) of transcripts in adaxial epidermal cells at 3h or 12 h > 0.45 (defined as being expressed) in all three replicate cotyledons in only adaxial epidermal cells; 2 fold change (log_2 FC) of > 1 with a false discovery rate (FDR) corrected P value < 0.05 calculated using Benjamini–Hochberg procedure (Benjamini et al., 2001): specifically between 0 to 3 h (UWL up-regulated transcripts); only expressed (RPKM>0.45 in all replicates) in 3 h (UWL specific transcripts); specifically between 3 to 12 h (WI up-regulated transcripts); only expressed (RPKM>0.45 in all replicates) in 12 h (WI specific transcripts); or sustained up-regulation from 0 to 3 h to 12 h (UWL/WI shared no change transcripts) of cotyledon culture. RPKM values in both adaxial epidermal and storage parenchyma cells are means ± SE of 3 replicate batches of cotyledons. Bases for rejecting these genes were: (1) Log_2FC < 1; (2) RPKM < 1; (3) Uniform wall layer deposition is actin independent; (4) Uniform wall layer deposition is Ca^{2+} independent; (5) Not a polarized vesicle trafficking candidate.

Sequence ID	Annotation	Reason for rejection	Relative transcript level (RPKM) in:						Log2 fold change during:			
			Adaxial epidermal cells at:			Storage parenchyma cells at:			Adaxial epidermal cells		Storage parenchyma cells	
			0 h	3 h	12 h	0 h	3 h	12 h	0 to 3 h	3 to 12 h	0 to 3 h	3 to 12 h
**UWL up-regulated/specific transcripts**												
Actin												
CL2210.C1	*VfActin7*	3	0.0 ± 0.0	1.3 ± 0.4	0.5 ± 0.2	0.0 ± 0.0	0.4 ± 0.2	0.5 ± 0.2	5.0*	-1.4	3.6	0.3
U8030	*VfActin12*	2	0.1 ± 0.1	0.9 ± 0.2	0.3 ± 0.2	0.0 ± 0.0	0.4 ± 0.4	0.3 ± 0.2	3.0*	-1.5	10.0	-0.6
Vesicle trafficking												
Exocytosis												
CL6200.C1	*Vf(Myosin)XI*	3	0.9 ± 0.3	3.1 ± 0.4	0.6 ± 0.1	0.0 ± 0.0	0.1 ± 0.1	0.0 ± 0.0	1.7*	-2.3*	-10.0	0.0
U21725	*VfAGD3*	1	4.7 ± 2.9	3.2 ± 1.3	3.9 ± 2.4	0.1 ± 0.0	1.2 ± 1.1	0.1 ± 0.1	-0.5	0.3	3.9	-3.6
U18006	*VfAGD12*	4	0.3 ± 0.1	4.6 ± 1.3	1.6 ± 0.4	0.2 ± 0.0	0.1 ± 0.0	0.3 ± 0.2	3.7*	-1.5*	-0.4	1.1
U4133	*VfGDI*	2	0.3 ± 0.2	0.9 ± 0.3	0.1 ± 0.1	0.1 ± 0.1	0.3 ± 0.1	0.0 ± 0.0	1.5	-3.1*	1.6	-10.0
U35180	*VfHMG-I*	2	0.7 ± 0.1	0.7 ± 0.1	0.7 ± 0.2	0.1 ± 0.0	0.4 ± 0.2	0.2 ± 0.1	0.1	0.0	1.5	-0.8
U9284	*VfPVA21 like*	1	2.1 ± 0.6	2.9 ± 0.4	2.0 ± 0.7	1.9 ± 0.4	2.1 ± 0.2	2.9 ± 0.2	0.4	-0.5	0.1	0.4
Endocytosis												
U12722	*VfDRP1C*	1	1.4 ± 0.6	2.2 ± 0.5	1.3 ± 0.4	0.1 ± 0.1	0.0 ± 0.0	0.0 ± 0.0	0.7	-0.7	-1.3	-0.1

Sequence ID	Annotation	Reason for rejection	Relative transcript level (RPKM) in: Adaxial epidermal cells at: 0 h	3 h	12 h	Storage parenchyma cells at: 0 h	3 h	12 h	Log2 fold change during: Adaxial epidermal cells 0 to 3 h	3 to 12 h	Storage parenchyma cells 0 to 3 h	3 to 12 h
**WI up-regulated/specific transcripts**												
Actin depolymerizing proteins												
CL7887.C2	*VfVLN4*	2	0.6 ± 0.2	0.3 ± 0.0	0.5 ± 0.1	0.3 ± 0.1	0.6 ± 0.1	0.3 ± 0.1	-1.0	0.7	0.9	-0.7
Vesicle trafficking												
Exocytosis												
U9470, U9471	*VfSYP112*	1	1.4 ± 0.6	0.4 ± 0.1	1.0 ± 0.2	0.2 ± 0.1	0.3 ± 0.1	0.3 ± 0.1	-1.8*	1.4	1.0	0.1
Endocytosis												
CL3098.C3	*VfDRP2B*	2	0.2 ± 0.1	0.5 ± 0.1	0.4 ± 0.2	0.5 ± 0.2	0.5 ± 0.3	1.0 ± 0.4	0.9	-0.1	0.0	1.1
**UWL/WI shared no change transcripts**												
Vesicle trafficking												
Exocytosis												
CL6109.C2	*VfSAR1C*	5	14.8 ± 2.1	33.7 ± 1.2	18.9 ± 1.1	0.4 ± 0.1	0.7 ± 0.5	0.5 ± 0.4	1.2*	-0.8	0.6	-0.5
CL4945.C3	*VfERV14*	1	1.0 ± 0.2	1.3 ± 0.1	1.7 ± 0.2	0.9 ± 0.2	0.8 ± 0.1	2.1 ± 0.7	0.4	0.4	-0.1	1.3
U19005	*VfSPPL3*	1	1.2 ± 0.1	1.0 ± 0.1	1.6 ± 0.2	0.6 ± 0.2	1.1 ± 0.1	1.5 ± 0.1	-0.3	0.7	0.8	0.5
U7624	*VfSYTF4*	2	0.1 ± 0.1	0.7 ± 0.2	0.3 ± 0.1	0.0 ± 0.0	0.4 ± 0.1	0.0 ± 0.0	3.0*	-1.3	10.0	-3.1

Table S4. *Statistical verification of spatial association between short, thin actin bundles and wall ingrowth papillae in adaxial epidermal cells of* V. faba *cotyledons.*

The R package spatstat was used to simulate percentage of wall ingrowth papillae associated with end(s) of short, thin actin bundles when the latter is laid down randomly in the adaxial epidermal cells. The distribution of the percent associations from 1000 random simulations was used to provide a reference against which to compare the data collected from cells co-stained with Congo Red to stain wall ingrowth papillae and Alexa-488 phalloidin to visualize actin to assess levels of significance (see Materials and methods for more details) for percentage of wall ingrowth papillae in measured cells. For each cell the mean ± standard deviation from 1000 simulations are shown.

	% Associated papillae in double stained cells	% Associated papillae in simulated cells Mean ± SD	% Associated papillae in simulated cells 99.5 percentile	Single tail p value for double stained cells
Cell 1	67	23 ± 9	43	< 0.001
Cell 2	61	20 ± 8	39	< 0.001
Cell 3	52	23 ± 8	40	0.004
Cell 4	63	18 ± 7	33	< 0.001
Cell 5	50	15 ± 7	29	< 0.001
Cell 6	56	20 ± 8	36	< 0.001
Cell 7	68	23 ± 8	40	< 0.001
Cell 8	65	19 ± 8	35	< 0.001
Cell 9	59	17 ± 6	30	< 0.001
Cell 10	59	22 ± 8	38	< 0.001
Cell 11	60	24 ± 7	37	< 0.001
Cell 12	57	21 ± 9	39	0.001
Cell 13	68	21 ± 8	36	< 0.001
Cell 14	50	20 ± 8	36	< 0.001
Cell 15	65	22 ± 9	40	< 0.001

Note in all cases the % associated papillae in double stained cells was greater than the upper tail (at the 99.5 percentile level) of the reference distribution of randomly generated associations. The p values quantify this further. This is strong support that the observed percent associations are very unlikely to have come about by chance and represent real associations. The assessment of these 15 cells suggests that the same would be expected for the other cells not assessed by this random approach.

Also note the exact values of the percentiles and p values in the table depend on the sequence of random numbers used in the simulations and will only be replicated exactly on the first run in the software when the random seed is initialized to the value in the code R code. Additional runs will lead to slightly different values for some cells but the overriding pattern is of very low p values across the 15 cells.

Original codes from The R software package (R Core Team, 2016, V3.3.1) with the library spatstat (Baddeley et al., 2015) to simulate percentage of wall ingrowth papillae associated with end(s) of short, thin actin bundles when the latter is laid down randomly in the adaxial epidermal cells.

```
# Perform simulations using random placement of actin bundles against locations of papillae for 15 cells
# to determine reference distributions of proportion of papillae that have contacts with actin bundles.
# Compare with actual proportion of contacts for each cell and determine p values
# K. Colyvas: Jun-2017
```

```
# The data sets have been embedded in the code (rather than read from files as in the orginal) to simplify
# the use for a reader wanting to replicate and/or check the code

library(spatstat)
library(lattice)
setwd("g:")

# Read the measured papillae coordinates
#p1=read.table("papillae all cells.txt", header = T)
# Save data file as R code for placement below so reader does not need to download separate files to run the code
#dput(p1)

p1=structure(list(cell = c(1L, 1L, 1L, 1L, 1L, 1L, 1L, 1L, 1L, 1L,
1L, 1L, 1L, 1L, 1L, 1L, 1L, 1L, 1L, 1L, 1L, 2L, 2L, 2L, 2L, 2L,
2L, 2L, 2L, 2L, 2L, 2L, 2L, 2L, 2L, 2L, 2L, 2L, 2L, 2L, 2L, 2L,
2L, 2L, 3L, 3L, 3L, 3L, 3L, 3L, 3L, 3L, 3L, 3L, 3L, 3L, 3L, 3L,
3L, 3L, 3L, 3L, 3L, 3L, 3L, 3L, 3L, 3L, 3L, 4L, 4L, 4L, 4L, 4L,
4L, 4L, 4L, 4L, 4L, 4L, 4L, 4L, 4L, 4L, 4L, 4L, 4L, 4L, 4L, 4L,
4L, 4L, 4L, 4L, 4L, 4L, 5L, 5L, 5L, 5L, 5L, 5L, 5L, 5L, 5L, 5L,
5L, 5L, 5L, 5L, 5L, 5L, 5L, 5L, 5L, 5L, 5L, 5L, 5L, 5L, 5L, 5L,
5L, 5L, 6L, 6L, 6L, 6L, 6L, 6L, 6L, 6L, 6L, 6L, 6L, 6L, 6L, 6L,
6L, 6L, 6L, 6L, 6L, 6L, 6L, 6L, 6L, 6L, 6L, 7L, 7L, 7L, 7L, 7L,
7L, 7L, 7L, 7L, 7L, 7L, 7L, 7L, 7L, 7L, 7L, 7L, 7L, 7L, 7L, 7L,
7L, 7L, 7L, 7L, 8L, 8L, 8L, 8L, 8L, 8L, 8L, 8L, 8L, 8L, 8L, 8L,
8L, 8L, 8L, 8L, 8L, 8L, 8L, 8L, 9L, 9L, 9L, 9L, 9L, 9L, 9L, 9L,
9L, 9L, 9L, 9L, 9L, 9L, 9L, 9L, 9L, 9L, 9L, 9L, 9L, 9L, 9L, 9L,
9L, 9L, 9L, 9L, 9L, 9L, 9L, 9L, 9L, 9L, 9L, 9L, 9L, 10L, 10L,
10L, 10L, 10L, 10L, 10L, 10L, 10L, 10L, 10L, 10L, 10L, 10L, 10L,
10L, 10L, 10L, 10L, 10L, 10L, 10L, 10L, 10L, 10L, 10L, 10L, 10L,
10L, 11L, 11L, 11L, 11L, 11L, 11L, 11L, 11L, 11L, 11L, 11L, 11L,
11L, 11L, 11L, 11L, 11L, 11L, 11L, 11L, 11L, 11L, 11L, 11L, 11L,
11L, 11L, 11L, 11L, 11L, 12L, 12L, 12L, 12L, 12L, 12L, 12L, 12L,
12L, 12L, 12L, 12L, 12L, 12L, 12L, 12L, 12L, 12L, 12L, 12L, 12L,
12L, 12L, 13L, 13L, 13L, 13L, 13L, 13L, 13L, 13L, 13L, 13L, 13L,
13L, 13L, 13L, 13L, 13L, 13L, 13L, 13L, 13L, 13L, 13L, 13L, 13L,
13L, 14L, 14L, 14L, 14L, 14L, 14L, 14L, 14L, 14L, 14L, 14L, 14L,
14L, 14L, 14L, 14L, 14L, 14L, 14L, 14L, 14L, 14L, 15L, 15L, 15L,
15L, 15L, 15L, 15L, 15L, 15L, 15L, 15L, 15L, 15L, 15L, 15L, 15L,
15L, 15L, 15L, 15L), X = c(22L, 17L, 15L, 61L, 62L, 36L, 96L,
126L, 107L, 147L, 165L, 179L, 197L, 215L, 257L, 321L, 344L, 370L,
397L, 296L, 352L, 20L, 44L, 58L, 72L, 74L, 98L, 132L, 117L, 164L,
177L, 194L, 215L, 204L, 253L, 239L, 272L, 274L, 296L, 362L, 379L,
395L, 398L, 391L, 5L, 18L, 41L, 45L, 64L, 34L, 98L, 109L, 126L,
142L, 125L, 191L, 234L, 210L, 232L, 271L, 293L, 278L, 270L, 336L,
368L, 380L, 374L, 387L, 342L, 6L, 62L, 59L, 101L, 95L, 87L, 135L,
119L, 155L, 170L, 186L, 268L, 279L, 263L, 269L, 263L, 293L, 313L,
311L, 333L, 339L, 360L, 368L, 345L, 378L, 396L, 387L, 10L, 14L,
44L, 42L, 70L, 99L, 117L, 133L, 127L, 164L, 157L, 178L, 184L,
204L, 212L, 232L, 280L, 294L, 290L, 287L, 324L, 342L, 367L, 377L,
375L, 364L, 389L, 391L, 21L, 6L, 40L, 54L, 57L, 85L, 95L, 108L,
114L, 158L, 163L, 151L, 167L, 155L, 178L, 220L, 242L, 306L, 334L,
334L, 366L, 362L, 372L, 353L, 389L, 25L, 42L, 67L, 64L, 75L,
91L, 128L, 134L, 155L, 169L, 186L, 194L, 193L, 206L, 211L, 226L,
251L, 293L, 301L, 319L, 332L, 339L, 343L, 347L, 351L, 24L, 28L,
```

```
70L, 64L, 50L, 86L, 115L, 126L, 136L, 127L, 169L, 183L, 153L,
205L, 191L, 219L, 286L, 301L, 263L, 322L, 106L, 98L, 112L, 93L,
92L, 100L, 144L, 142L, 141L, 163L, 170L, 162L, 189L, 180L, 197L,
208L, 238L, 231L, 242L, 246L, 236L, 227L, 239L, 256L, 253L, 274L,
272L, 268L, 273L, 276L, 290L, 288L, 316L, 329L, 344L, 355L, 361L,
11L, 16L, 41L, 43L, 73L, 98L, 117L, 127L, 136L, 152L, 157L, 171L,
165L, 167L, 185L, 204L, 214L, 225L, 256L, 282L, 290L, 291L, 287L,
313L, 326L, 347L, 368L, 365L, 379L, 35L, 18L, 43L, 26L, 57L,
60L, 72L, 92L, 99L, 112L, 123L, 136L, 153L, 166L, 157L, 167L,
178L, 192L, 206L, 234L, 228L, 226L, 241L, 252L, 267L, 297L, 292L,
316L, 331L, 337L, 45L, 39L, 47L, 70L, 77L, 85L, 127L, 144L, 171L,
165L, 189L, 204L, 212L, 222L, 207L, 223L, 220L, 237L, 258L, 278L,
267L, 317L, 314L, 17L, 11L, 35L, 49L, 65L, 63L, 79L, 96L, 128L,
142L, 169L, 184L, 177L, 192L, 199L, 216L, 212L, 250L, 244L, 269L,
289L, 331L, 344L, 357L, 352L, 3L, 6L, 31L, 27L, 106L, 115L, 126L,
127L, 159L, 196L, 211L, 241L, 233L, 258L, 269L, 307L, 305L, 327L,
349L, 359L, 358L, 379L, 14L, 42L, 35L, 35L, 61L, 65L, 111L, 122L,
149L, 157L, 196L, 226L, 211L, 236L, 241L, 286L, 331L, 362L, 378L,
388L), Y = c(14L, 41L, 74L, 16L, 40L, 75L, 31L, 69L, 74L, 74L,
24L, 54L, 52L, 73L, 78L, 70L, 47L, 54L, 56L, 40L, 36L, 19L, 51L,
24L, 32L, 70L, 27L, 22L, 70L, 46L, 61L, 71L, 56L, 82L, 28L, 50L,
36L, 64L, 13L, 30L, 57L, 22L, 38L, 59L, 33L, 59L, 3L, 19L, 51L,
90L, 32L, 47L, 20L, 74L, 97L, 89L, 14L, 45L, 62L, 24L, 47L, 60L,
73L, 8L, 36L, 45L, 52L, 88L, 99L, 61L, 38L, 67L, 64L, 79L, 90L,
35L, 76L, 61L, 48L, 59L, 13L, 13L, 38L, 46L, 69L, 49L, 11L, 40L,
27L, 43L, 18L, 50L, 76L, 5L, 48L, 73L, 59L, 70L, 53L, 84L, 46L,
54L, 26L, 14L, 56L, 24L, 85L, 21L, 48L, 9L, 65L, 9L, 12L, 21L,
46L, 76L, 28L, 54L, 22L, 24L, 68L, 83L, 77L, 89L, 14L, 69L, 19L,
46L, 99L, 5L, 14L, 53L, 55L, 92L, 11L, 11L, 54L, 72L, 91L, 40L,
79L, 20L, 38L, 71L, 41L, 55L, 70L, 81L, 30L, 70L, 40L, 30L, 70L,
82L, 73L, 66L, 83L, 34L, 11L, 60L, 77L, 85L, 70L, 94L, 68L, 53L,
25L, 65L, 26L, 37L, 22L, 66L, 80L, 11L, 46L, 68L, 19L, 50L, 85L,
68L, 20L, 18L, 57L, 95L, 15L, 19L, 80L, 51L, 95L, 73L, 18L, 21L,
64L, 69L, 24L, 33L, 37L, 59L, 92L, 68L, 1L, 32L, 63L, 11L, 66L,
48L, 40L, 58L, 71L, 8L, 6L, 13L, 22L, 38L, 58L, 69L, 96L, 39L,
56L, 18L, 26L, 54L, 69L, 80L, 69L, 80L, 94L, 92L, 54L, 60L, 88L,
65L, 74L, 53L, 89L, 65L, 58L, 29L, 60L, 18L, 43L, 89L, 91L, 26L,
49L, 53L, 12L, 68L, 15L, 9L, 14L, 28L, 49L, 81L, 17L, 33L, 59L,
25L, 87L, 72L, 25L, 62L, 55L, 69L, 97L, 39L, 61L, 87L, 45L, 73L,
90L, 26L, 36L, 46L, 54L, 69L, 35L, 59L, 80L, 4L, 46L, 69L, 73L,
47L, 85L, 42L, 70L, 67L, 91L, 15L, 12L, 23L, 69L, 1L, 64L, 6L,
6L, 83L, 18L, 38L, 13L, 34L, 47L, 62L, 70L, 60L, 97L, 68L, 69L,
61L, 92L, 63L, 87L, 13L, 37L, 25L, 33L, 59L, 73L, 71L, 61L, 64L,
73L, 44L, 30L, 59L, 60L, 18L, 17L, 25L, 30L, 60L, 50L, 35L, 13L,
22L, 48L, 73L, 72L, 96L, 53L, 60L, 71L, 27L, 51L, 97L, 24L, 6L,
17L, 58L, 82L, 41L, 9L, 31L, 57L, 12L, 15L, 7L, 86L, 46L, 23L,
48L, 64L, 79L, 22L, 92L, 56L, 89L, 18L, 54L, 4L, 17L, 30L, 53L,
91L, 73L, 2L, 5L, 44L, 70L)), .Names = c("cell", "X", "Y"), class = "data.frame", row.names = c(NA,
-380L))

head(p1)

# Plot papillae locations
# xyplot(Y~X|factor(cell), data=p1)
```

```
# Prepare table of numbers of papillae counted per cell
num.papillae=as.data.frame(table(p1$cell))
names(num.papillae)=c("cell","n")
head(num.papillae)
num.cells=nrow(num.papillae)

# Read actual numbers of papillae associated with bundles – measured from double-stained cells
# produced later in response to reviewer and added for convenience rather than change the code above
#p.associated=read.table("Papillae all cells - prop associated.txt", header = T)
#dput(p.associated)

p.associated=structure(list(cell = 1:15, n.papillae = c(21L, 23L, 25L, 27L,
28L, 25L, 25L, 20L, 37L, 29L, 30L, 23L, 25L, 22L, 20L), n.papillae.associated = c(14L,
14L, 13L, 17L, 14L, 14L, 17L, 13L, 22L, 17L, 18L, 13L, 17L, 11L,
13L), prop.papillae.associated = c(0.666666667, 0.608695652,
0.52, 0.62962963, 0.5, 0.56, 0.68, 0.65, 0.594594595, 0.586206897,
0.6, 0.565217391, 0.68, 0.5, 0.65)), .Names = c("cell", "n.papillae",
"n.papillae.associated", "prop.papillae.associated"), class = "data.frame", row.names = c(NA,
-15L))

head(p.associated)

# Read the measured actin bundle data
#ab1=read.table("actin bundles all cells.txt", header = T)
#dput(ab1)

ab1=structure(list(cell = 1:15, num.bundles = c(47L, 42L, 51L, 36L,
29L, 43L, 46L, 38L, 36L, 46L, 50L, 47L, 42L, 42L, 47L). Names = c("cell", "num.bundles"), class = "data.frame",
row.names = c(NA,
-15L))

head(ab1)

# Initialise the matrix to store the distribution summaries from the random actin bundle assignmentsfor each cell
quantiles.cell=matrix(NA,ncol=9,nrow=num.cells)
dimnames(quantiles.cell)=list(NULL,c("cell","mean","SD", "0.5%","2.5%","median","97.5%","99.5%","max"))
#head(quantiles.cell)

# Matrix for counts of simulations that had proportion of random contacts between actin bundles
# and papillae >= observed proportion
exceed.or.equal=matrix(NA,ncol=3,nrow=num.cells)
dimnames(exceed.or.equal)=list(NULL,c("cell","number.exceed.or.equal","p.value"))

# Open file to receive 4 plots for each cell
pdf(file="Papillae spatial plots.pdf")

set.seed(1234567)

for (i in 1:num.cells) {
num.pap=num.papillae$n[i]
num.bundles=ab1$num.bundles[i]

# make ppp object for papillae
P=with(p1[p1$cell==i,], ppp(X,Y,c(-20,420),c(0,110)))
```

```
# Generate random actin bundles
J= 1000 # number of random simulations

# Initialise the matrix to receive the random simulations
ab.random = matrix(NA,ncol = 5,nrow=J)
dimnames(ab.random)=list(NULL, c("cell","num.bundles.close.r","num.pap.close.r","num.pap","p.close.r"))

# Determine the distribution for proportion of papillae with contacts with actin bundles for a cell under random
placement
for (j in 1:J) {
# Offsets and scale factors used in case they were neeed for rescaling - decided against it but code kept
x.offset = 0
y.offset = 0
x.scale = 400
y.scale = 100

# The first end of each randomly placed bundle
x1=runif(num.bundles)*x.scale + x.offset
y1=runif(num.bundles)*y.scale + y.offset

# Add the second end of each randomly placed first end of a bundle in accord with observed
# relationship between ends from extra measurements made on cell 1
# Distance apart normally distributed mean=16.0, SD=2.7
# Angles found to be randomly distributed from extra measurements on cell 1
# Additional measurement data and code provided at the end of the code for the random simulations
angle=runif(num.bundles)*360
bundle.length=rnorm(num.bundles,16,2.7)
x2=x1+bundle.length*cos(angle/180*pi)
y2=y1+bundle.length*sin(angle/180*pi)

# Make a ppp object from the randomly generated actin bundles
end1=cbind(x1,y1)
end2=cbind(x2,y2)
end12=as.data.frame(rbind(end1,end2))
#plot(y1~x1,data=end12)
ABPr = ppp(end12$x1,end12$y1,window=owin(xrange=c(-50,500),yrange=c(-50,150)))
# plot(ABPr)

# Determine distances between papillae and ends of random actin bundles to decide if there is "touching" contact
dr <- crossdist(P,ABPr)
#hist(dr,nclass=30)

# Count how many bundles are "touching" the papillae (within 6 pixels ~ 400 nm)
dist.bundles.close.r=dr[dr<=6]
num.bundles.close.r=length(dist.bundles.close.r)

# Count how many papillae have actin bundles touching
# need to correct for the possibility that multiple bundles are touching a single pappila

papillae.close.r=matrix(NA,ncol=1,nrow=num.pap)
dimnames(papillae.close.r)=list(NULL,"papillae.close.r")

for (k in 1:num.pap) {
if (length(dr[k,dr[k,]<=6])>0) papillae.close.r[k]=1  else papillae.close.r[k]=0
}
```

```
# Proportion of papillae with 1 or more bundles "touching"
num.papillae.close.r=sum(papillae.close.r)
p.close.r=num.papillae.close.r/num.pap

ab.random[j,1]=i
ab.random[j,2]=num.bundles.close.r # number of actin bundles close to papillae
ab.random[j,3]=num.papillae.close.r # number of papillae with 1 or more actin bundles close
ab.random[j,4]=num.pap
ab.random[j,5]=p.close.r
#head(ab.random)
}

# Plot summary information for each cell
op=par(mfrow=c(2,2))
plot(P,main=paste("WI Papillae (X,Y) locations, Cell",i))

# The reference distribution from random simulation for each cell and the vertical line is the actual proportion
hist(ab.random[,"p.close.r"],nclass=80,xlim=c(0,0.80),main=paste("Random & actual, Cell ",i),xlab="Proportion
papillae <=6 pixels from 1 an actin bundles")
abline(v=p.associated$prop.papillae.associated[i],lwd=3)

# Confirming there are some multiple actin bundle contacts per papillae and validating the decision to
# correct for this in the reference distribution determination

# Create a little random noise so when plotted the overlap of many plotted points is more obvious
jitter=.4
YY=ab.random[,"num.bundles.close.r"] + runif(J)*jitter-jitter/2
XX=ab.random[,"num.pap.close.r"] + runif(J)*jitter-jitter/2

plot(YY~XX,main=paste("Compare random contacts, Cell ",i),
    xlab="n(papillae with 1 or more bundles close)",
    ylab="n(bundles close to pappilae)")
abline(0,1)
par(op)

quantiles.cell[i,1]=i
quantiles.cell[i,2]=mean(ab.random[,"p.close.r"])
quantiles.cell[i,3]=sd(ab.random[,"p.close.r"])
quantiles.cell[i,4:8]=quantile(ab.random[,"p.close.r"],probs=c(.005,.025,.5,.975,.995))
quantiles.cell[i,9]=max(ab.random[,"p.close.r"])

# number of proportions >= observed % association and 1 sided p value
exceed.or.equal[i,1] = i
exceed.or.equal[i,2] =
length(ab.random[,"p.close.r"][ab.random[,"p.close.r"]>=p.associated[i,"prop.papillae.associated"]])
exceed.or.equal[i,3] = exceed.or.equal[i,2]/J
}
dev.off()

#head(quantiles.cell)

# Summary plot comparing random and actual across all cells

pdf(file="Significance of effects.pdf")
```

```
op=par(mfrow=c(3,1))
plot(prop.papillae.associated~cell, data=p.associated,ylim=c(0,1),pch=16,main="Random probability limits 0.5%
to 99.5%, about the median")
points(quantiles.cell[,"median"]~quantiles.cell[,"cell"],pch=3)
arrows(quantiles.cell[,"cell"],quantiles.cell[,"median"],quantiles.cell[,"cell"],quantiles.cell[,"99.5%"],.05,90)
arrows(quantiles.cell[,"cell"],quantiles.cell[,"median"],quantiles.cell[,"cell"],quantiles.cell[,"0.5%"],.05,90)

plot(prop.papillae.associated~cell, data=p.associated,ylim=c(0,1),pch=16,main="Random probability limits 2.5%
to 97.5%, about the median")
points(quantiles.cell[,"median"]~quantiles.cell[,"cell"],pch=3)
arrows(quantiles.cell[,"cell"],quantiles.cell[,"median"],quantiles.cell[,"cell"],quantiles.cell[,"97.5%"],.05,90)
arrows(quantiles.cell[,"cell"],quantiles.cell[,"median"],quantiles.cell[,"cell"],quantiles.cell[,"2.5%"],.05,90)

plot(prop.papillae.associated~cell, data=p.associated,ylim=c(0,1),pch=16,main="Random probability limits 2.5%
to 97.5%, about the mean")
points(quantiles.cell[,"mean"]~quantiles.cell[,"cell"],pch=3)
arrows(quantiles.cell[,"cell"],quantiles.cell[,"median"],quantiles.cell[,"cell"],quantiles.cell[,"97.5%"],.05,90)
arrows(quantiles.cell[,"cell"],quantiles.cell[,"median"],quantiles.cell[,"cell"],quantiles.cell[,"2.5%"],.05,90)
par(op)

dev.off()

(table.results=cbind(as.data.frame(quantiles.cell),p.associated,as.data.frame(exceed.or.equal)))
write.table(table.results,file="Table of results.txt",sep="\t",row.names=F)

# Saved for review as part of testing what the simulation coded was doing
#write.table(ab.random,file="Cell 15 random results.txt",sep="\t",row.names=F)

#
==========================================================================================
# Additional code - used to determine actin bundle angle and length distributions for use in the
# random simulation of actin bundles in the code above

# Detailed measurements of actin bundles for cell 1 to determine the distribution of their angles and lengths

#ab=read.table("Cell 1 - actin bundles extra measurements.txt", header = T,sep="\t")
#dput(ab)
ab=structure(list(X0 = c(40L, 29L, 61L, 39L, 105L, 63L, 96L, 37L,
178L, 129L, 97L, 90L, 135L, 214L, 199L, 94L, 100L, 256L, 257L,
263L, 266L, 370L, 398L, 352L, 384L, 360L, 322L, 384L, 372L, 333L,
374L, 297L, 339L, 100L, 139L, 127L, 149L, 165L, 179L, 221L, 199L,
266L, 159L, 186L, 200L, 269L, 55L), Y0 = c(19L, 72L, 14L, 76L,
7L, 38L, 71L, 74L, 54L, 40L, 33L, 44L, 69L, 73L, 18L, 69L, 68L,
78L, 79L, 47L, 26L, 53L, 55L, 35L, 68L, 90L, 70L, 53L, 73L, 32L,
75L, 39L, 62L, 68L, 47L, 69L, 38L, 31L, 41L, 38L, 60L, 48L, 59L,
48L, 61L, 35L, 58L), X1 = c(32L, 14L, 78L, 48L, 115L, 59L, 109L,
43L, 185L, 147L, 83L, 94L, 150L, 232L, 209L, 107L, 121L, 261L,
264L, 272L, 273L, 389L, 412L, 352L, 397L, 369L, 336L, 398L, 385L,
338L, 379L, 299L, 324L, 117L, 151L, 142L, 153L, 174L, 178L, 234L,
208L, 275L, 166L, 202L, 214L, 276L, 62L), Y1 = c(13L, 73L, 9L,
88L, 20L, 51L, 75L, 87L, 39L, 47L, 47L, 28L, 54L, 73L, 26L, 74L,
61L, 89L, 66L, 57L, 40L, 41L, 47L, 54L, 78L, 78L, 80L, 54L, 72L,
44L, 62L, 22L, 75L, 62L, 32L, 66L, 54L, 24L, 56L, 30L, 47L, 58L,
73L, 51L, 52L, 43L, 43L), XM = c(39L, 10L, 65L, 44L, 110L, 61L,
```

```
102L, 40L, 181L, 138L, 90L, 92L, 142L, 223L, 204L, 101L, 110L,
258L, 260L, 267L, 270L, 380L, 405L, 352L, 390L, 365L, 329L, 391L,
379L, 336L, 377L, 298L, 331L, 108L, 145L, 135L, 151L, 170L, 178L,
227L, 204L, 270L, 163L, 194L, 207L, 273L, 59L), YM = c(15L, 72L,
12L, 82L, 14L, 45L, 73L, 71L, 46L, 43L, 40L, 36L, 61L, 73L, 22L,
72L, 64L, 84L, 72L, 52L, 33L, 47L, 51L, 44L, 73L, 84L, 75L, 53L,
73L, 38L, 69L, 30L, 69L, 65L, 40L, 68L, 46L, 37L, 48L, 34L, 54L,
53L, 66L, 49L, 56L, 39L, 50L), ANGLE = c(38L, 173L, 159L, 57L,
61L, 112L, 23L, 63L, 111L, 21L, 138L, 105L, 128L, 3L, 42L, 156L,
20L, 66L, 127L, 50L, 58L, 147L, 155L, 90L, 35L, 124L, 36L, 169L,
5L, 74L, 113L, 97L, 47L, 15L, 133L, 166L, 77L, 145L, 82L, 28L,
122L, 51L, 63L, 11L, 145L, 53L, 112L), LENGTH = c(13L, 15L, 18L,
17L, 18L, 14L, 16L, 14L, 17L, 19L, 19L, 16L, 23L, 18L, 13L, 12L,
23L, 12L, 15L, 14L, 15L, 21L, 17L, 19L, 16L, 16L, 17L, 15L, 13L,
15L, 13L, 17L, 16L, 19L, 19L, 16L, 17L, 12L, 15L, 15L, 17L, 13L,
16L, 16L, 16L, 10L, 16L), ANGLE.Binned = structure(c(4L, 3L,
3L, 4L, 5L, 6L, 1L, 5L, 6L, 1L, 2L, 6L, 2L, 1L, 4L, 3L, 1L, 5L,
2L, 4L, 4L, 2L, 3L, 6L, 4L, 2L, 4L, 3L, 1L, 5L, 6L, 6L, 4L, 1L,
2L, 3L, 5L, 2L, 5L, 1L, 2L, 4L, 5L, 1L, 2L, 4L, 6L), .Label = c("0 ù 30",
"120 ù 150", "150 ù 180", "30 ù 60", "60 ù 90", "90 ù 120"), class = "factor")), .Names = c("X0",
"Y0", "X1", "Y1", "XM", "YM", "ANGLE", "LENGTH", "ANGLE.Binned"
), class = "data.frame", row.names = c(NA, -47L))

head(ab)

# Make psp object for actin bundles
ABL = with(ab,psp(X0, Y0, X1, Y1,window=owin(xrange=c(0,420),yrange=c(0,100))))
plot(ABL,xlim=c(0,420))

# Are angles randomly distributed? YES.
hist(ab$ANGLE)
table(ab$ANGLE.Binned)

# Chi-sq test to see if all angle groups are randomly distributed, i.e equal in all ranges
o=as.data.frame(table(ab$ANGLE.Binned)) # observed
e=sum(o$Freq)/6 # expected
cbind(o,e)
chi.sq=sum((o$Freq-e)^2/e)
# p value for chi-sq test
1-pchisq(chi.sq,5)
# As p = .93 there is no evidence of a particular orientation being preferred so random angles
# were used in the simulations


# A normal distribution (Mean 16.0, SD 2.7) is a satisfactory model for the lengths of the
# actin bundles from cell 1. The model was used for the bundle lengths in the simulation above
hist(ab$LENGTH)
summary(ab$LENGTH)
densityplot(~ab$LENGTH)
qqmath(~ab$LENGTH)
shapiro.test(ab$LENGTH)

mean(ab$LENGTH)
sd(ab$LENGTH)
```

Computational Information S1. *Estimated portion of exocytosed plasma membrane retrieved by endocytosis during assembly of a wall ingrowth papilla*

1. ***Volume estimate of cell wall matrix material required to construct a papilla***
   Each wall ingrowth papilla is comprised of an outer sheath of callose enclosing an inner core of cellulose microfibrils embedded in matrix polysaccharides (Talbot *et al*., 2007; Vaughn *et al*., 2007).
   Since the membrane contribution of vesicles delivering cellulose and callose synthases cannot be accounted for, these are set aside. Hence the computation specifically addresses matrix material occupying the inner core volume using the following dimension of the inner core:
   (a) Radius (r) = 141 ± 12 nm (n = 100)
   (b) Length (l) = 380 ± 12 nm (n = 100)

   Assuming that the shape of the inner core approximates a cylinder, 50% of its volume (V) is given by:
   $$V_{mp} = (\pi r^2 l)/2$$
   That is: 23,743,584 nm^3

2. ***Number of vesicles required to deliver the matrix polysaccharide volume of a wall ingrowth papilla.***
   The volume of one vesicle carrying a cargo of matrix wall material was estimated using the radius of vesicle performing this function in root hairs (i.e., 33 nm; see Ketelaar *et al*., 2008). Assuming the vesicle approximates a sphere, its volume will be:
   $$V_v = (4\pi r^3)/3$$
   That is: 150,593 nm^3
   Thus the number (N) of vesicles required to deliver the matrix polysaccharide volume of a wall ingrowth papilla is:
   $$N = V_{mp}/V_v$$
   That is: 158 vesicles

3. ***Total surface area of vesicle exocytosed to deliver the matrix polysaccharide content of a wall ingrowth papilla***
   The total surface area of vesicles will be given by:
   $A_v = (4\pi r^2)N$
   That is: 2,163,065 nm^2

4. ***Surface area of a papilla modelled as a cylinder***
   The following dimensions of a papilla were used:
   Radius (r) = 215 ± 8 nm (n = 240)
   Length (l) = 519 ± 18 nm (n = 240)

   The surface area of the cylinder (and hence papilla, A_p) will be the sum of the side (A_s) and top (A_t) where:
   $$A_s = 2\pi rl$$
   $$A_t = \pi r^2$$
   That is: 846,669 nm^2

5. ***Portion of exocytosed membrane retrieved by endocytosis***
   Portion (%) of retrieved membrane = $[(A_v - A_p)/A_v] \times 100$

   That is: 61%
